# Shifts in vegetation impact estuary microbiomes

**DOI:** 10.1128/msystems.01071-26

**Published:** 2026-09-30

**Authors:** Georgina L. Aitolo, Ian M. Rambo, Rachel E. Weisend, Megan M. Mullis, Susannah Tringe, James C. Kosmopoulos, Karthik Anantharaman, Brandi Kiel Reese, Brett J. Baker, Valerie De Anda

**Affiliations:** 1 Department of Interdisciplinary Life Sciences, The University of Texas at Austin 12330, Austin, Texas, USA; 2 Department of Integrative Biology, The University of Texas at Austin 12330, Austin, Texas, USA; 3 Department of Marine Science, The University of Texas at Austin 12330, Austin, Texas, USA; 4 Genomics and Bioinformatics Research Unit, U.S. Department of Agriculture, Agricultural Research Service, Gainesville, Florida, USA; 5 Texas A&M University-Corpus Christi 14738 , Corpus Christi, Texas, USA; 6 Lawrence Berkeley National Laboratory, Department of Energy Joint Genome Institute 1666, Berkeley, California, USA; 7 Microbiology Doctoral Training Program, University of Wisconsin-Madison 5228, Madison, Wisconsin, USA; 8 Department of Bacteriology, University of Wisconsin-Madison 5228, Madison, Wisconsin, USA; 9 University of South Alabama 5557, Mobile, Alabama, USA; 10 Dauphin Island Sea Lab 2808, Dauphin Island, Alabama, USA; 11 Department of Functional and Evolutionary Ecology, University of Vienna 27258, Vienna, Austria; UiT Norges arktiske universitet Arctic Centre for Sustainable Energy, Tromsø, Norway

**Keywords:** microbes, metagenomics, mud island, mangrove, seagrass, marshes, coastal environment, viruses

## Abstract

Coastal wetlands, including mangrove-cordgrass mosaics, are among Earth’s most effective carbon stores sequestering >1 Pg C/year. Rapid sea level rise, warming, and storm intensification are now reshaping these habitats. Vegetation type influences root exudates, sediment redox profiles, and organic matter quality. However, the impacts of vegetation on microbe–virus networks that mediate carbon burial and nutrient cycling remain unclear. Here, we sampled coastal sediment profiles in patches of cordgrass (dominated by *Sporobolus alterniflorus*), black mangrove (*Avicennia germinans*), and seagrass on the Texas Gulf Coast. We obtained 491 bacterial and archaeal metagenome assembled genomes (MAGs) and 1,097 viral MAGs (vMAGs) from 55 surface sediment samples spanning day and night during summer and fall. Phylogenetic and comparative analyses revealed dominant lineages across the distinct vegetation types, organized into metabolic guilds based on similar protein compositions, revealing contrasting and complementary sulfur, iron, and nitrogen cycling pathways that regulate greenhouse gas emissions and encode genes important for carbon metabolism. Inference from virus–host linkages revealed that dominant lineages were infected, and viral communities have genes for organic carbon degradation, potentially shaping the microbial community dynamics in this ecosystem. This spatio-temporal characterization of estuary ecotone microbiomes provides a framework to better understand the diversity and metabolism of these productive coastal ecosystems.

**IMPORTANCE:**

Coastal wetlands are among the most effective environments for storing carbon and regulating climate. As sea level rise and changing weather patterns drive shifts in coastal vegetation, these vulnerable ecosystems are undergoing rapid transformation. Yet, little is known about the microbes that regulate carbon storage and nutrient cycling. Here, we characterized microbial and viral communities associated with coastal wetland sediments along the Texas Gulf Coast. We identified key microbial groups responsible for key carbon, sulfur, nitrogen, and iron cycling and showed that vegetation type influences their distribution and ecological roles. We also found that viruses interact with dominant microbial groups and carry genes linked to carbon processing, suggesting an important role in shaping ecosystem function. These findings provide insights into the mechanisms that sustain coastal wetlands and help predict how shifts in vegetation may affect nutrient cycling and ecosystem resilience in the future.

## INTRODUCTION

Coastal mangroves and marshes are productive wetland ecosystems at the interface of terrestrial and marine environments, with high turnover rates of carbon and nutrients mediated by microbial communities (1, 2). Despite low occupancy of mangroves, salt marshes, and seagrasses (covering 0.2–0.5% of the coastal ocean) (3, 4), these vegetated environments are efficient natural sinks of carbon (5), contributing ~10–15% (18.4–34.4 Tg C year^−1^) of carbon stored in coastal sediments, referred to as “blue carbon” burial (3). A significant portion of net production in estuaries (~200 Tg C year^−1^) (6) is lost or recycled to the atmosphere as CO­_2_ (34.1 Tg year^−1^, ~20%) or exported to the ocean (117.9 Tg C year^−1^, ~60%) (7). Natural wetland environments, including mangrove forests, are estimated to be the largest natural methane source on Earth (8), contributing about 15–45% of global annual methane emissions (9).

The reduced frequency of winter hard freeze events (≤−2°C for an extended period) due to climate change is allowing for the northward expansion of black mangroves (*A. germinans*) in subtropical regions such as the northeast Florida coast (10) and the Texas coast (11). Populations of *A. germinans* have existed along the Texas coast for hundreds of years (12) although most wetlands in the Gulf of Mexico are dominated by *Sporobolus* species salt marshes, which were established 3,000–4,000 years ago (13). A potential cause of mangrove expansion in this region, in addition to milder winters, is the more effective allocation and conversion of nutrients into biomass by *A. germinans* versus *Sporobolus alterniflorus* (smooth cordgrass, hereafter called *Sporobolus*) in these ecotones (14), which is likely mediated by microbial nitrogen, sulfur, iron, and carbon cycling and tightly coupled microbe–nutrient–plant connections seen in mangrove soils (15).

**Fig 1:**
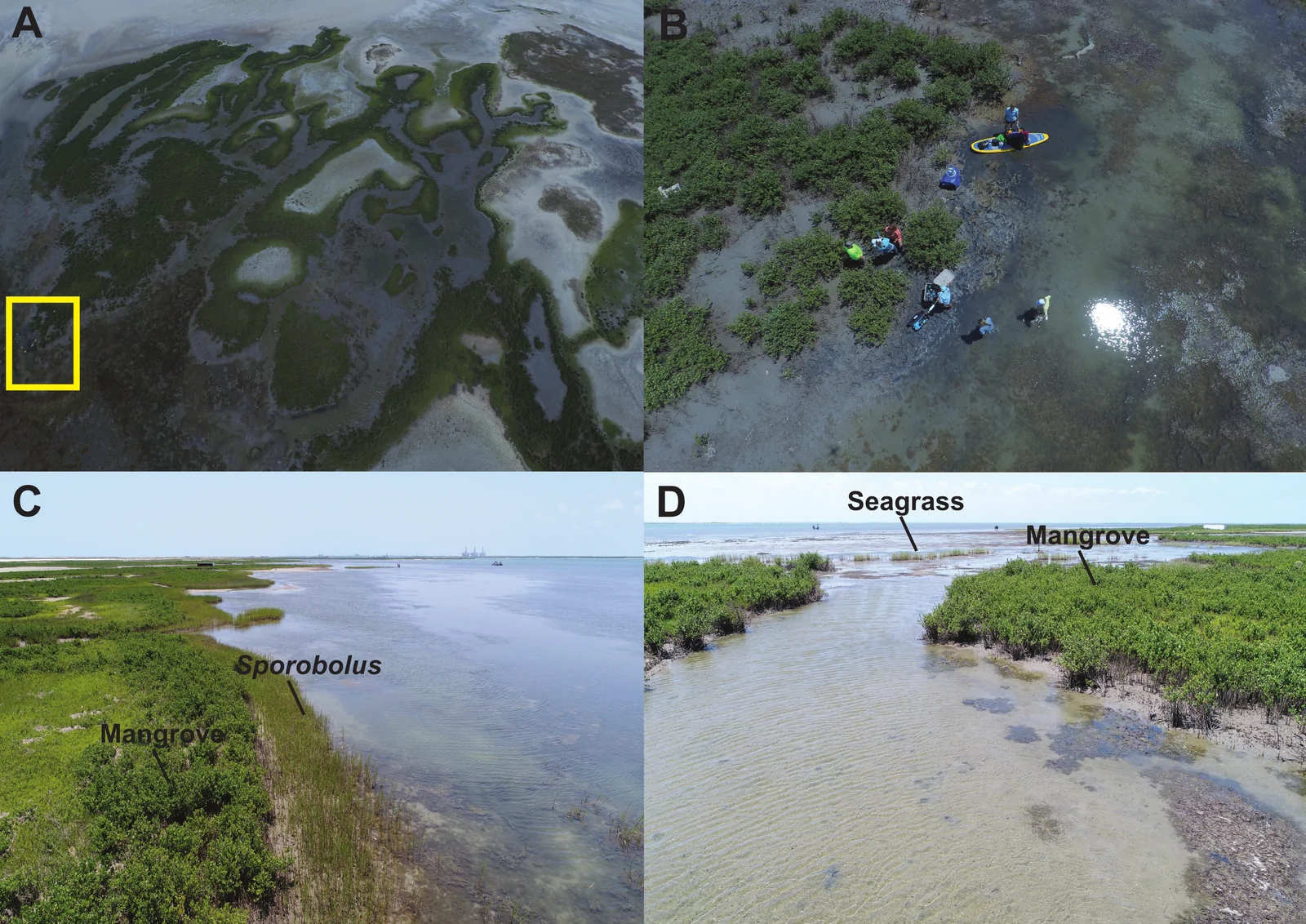
Aerial drone images of the Mud Island sampling site. Photos were taken on 6 June 2018. (**A**) Overview of the MANERR sampling area at Mud Island. The sampling site is indicated by the yellow square, with a zoomed-in view in panel **B**. (**C and D)** The geographical locations of the *S. alterniflorus* (*Sporobolus*) and *A. germinans* (mangrove) ecotone, with nearby submerged seagrass. Sampling coordinates are included in Table S1.

Mud Island (MI) within the Mission-Aransas National Estuarine Research Reserve (MANERR) represents a unique area to study mangrove expansion along the Texas coast. Mangrove populations at this site increased from 65 acres in 1980 to 1,534 acres in 2015 (16). At MI, *A. germinans* and *Sporobolus* cordgrass co-exist within an ecotone (Fig. 1). MI has been considered a vegetation transition zone site in the past (17) but has become mangrove dominated in recent years. Environmental conditions at MI are strongly seasonal, with freshwater inflow during the rainy season and high salinity during dry summer months. As an ecotone, MI presents a unique ecosystem; however, there is a lack of comprehensive documentation on the contribution of *A. germinans* (hereafter mangrove) and *Sporobolus* to blue carbon sequestration in this region (18). Also, the roles of microbial communities and functional capacity in this ecotone remain largely unexplored. Most microbial studies of other mangrove ecosystems have utilized cultivation and single-gene surveys of diversity (19–25). The biogeochemical roles of microbes in mangrove sediments have only been recently investigated via metagenomics (26–28). These studies revealed extensive novel diversity and a broad repertoire of carbon and nutrient metabolisms (27, 28). Within this ecosystem, viruses also act as key regulators of microbial community structure and function. By infecting and lysing host cells, viruses influence bacterial population, promote assemblage (29), and regulate the abundance (30) of dominant bacteria in the deep biosphere (31). As a result, they exert substantial influence over both local and global biogeochemical cycles in coastal mangrove ecosystems (27, 32).

Here, we characterize the microbial communities and microbe–virus interactions within Mud Island mangrove-cordgrass ecotones. This study provides new insights into microbial diversity and shared metabolic capabilities in coastal sediments, thereby advancing our understanding of key microbial communities involved in global nutrient cycling.

## RESULTS

### Microbial community composition and structure

**Fig 2:**
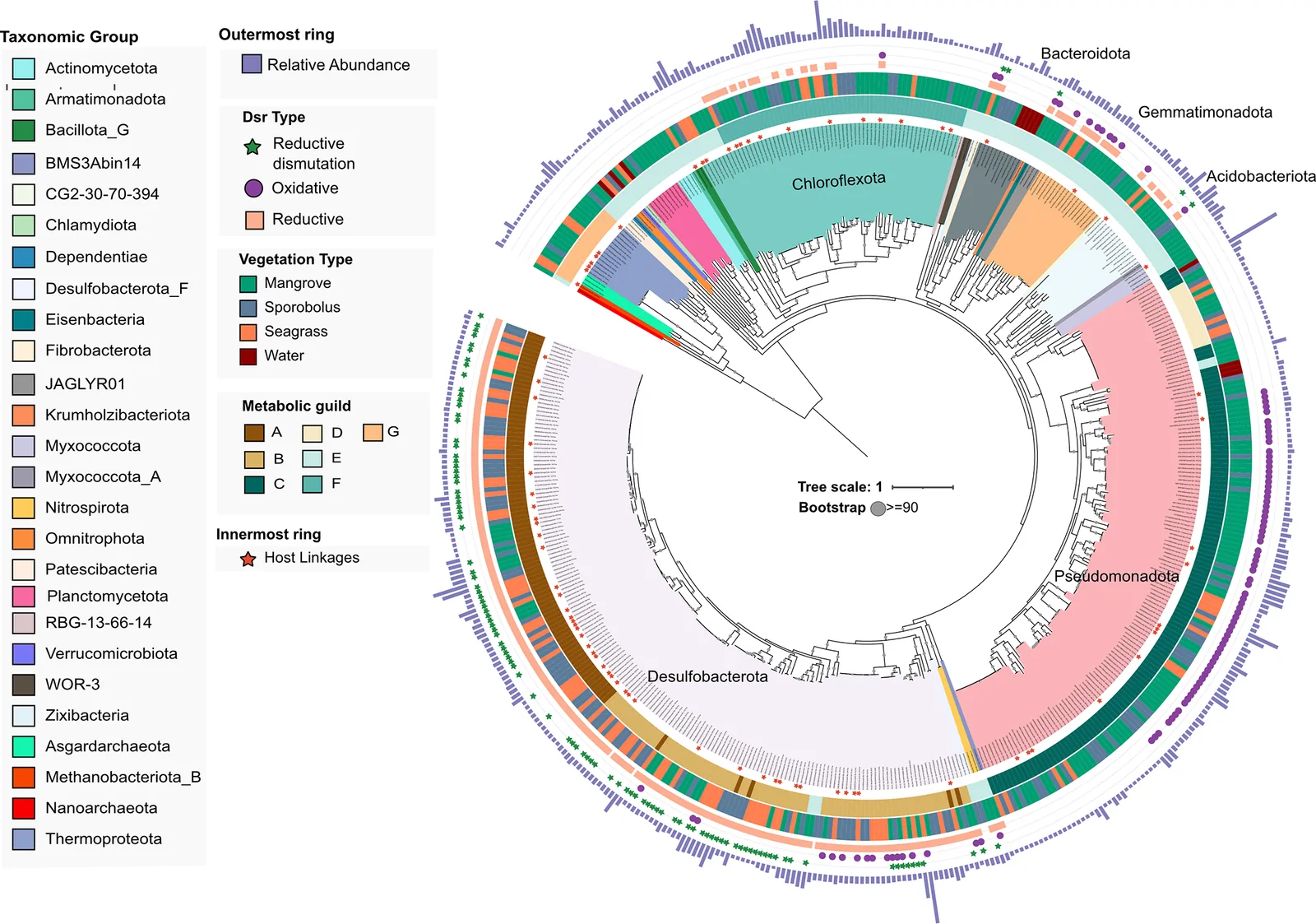
Taxonomic and metabolic diversity of 491 reconstructed bacterial and archaeal MAGs obtained from Mud Island sediments. From inner to outer visual layers, the tree is annotated to follow the progression (1) phylogenetic tree → (2) bootstraps → (3) taxonomic affiliation → (4) virus–host linkage → (5) metabolic guild → (6) vegetation → (7) Dsr type → (8) relative abundance. (1) A maximum-likelihood phylogenetic tree was inferred from concatenated alignments of 37 conserved ribosomal proteins (RP) extracted with PhyloSift (33) and built using IQ-TREE v2.1.4. The best-fitting substitution model was selected with ModelFinder Plus, and branch support was evaluated using ultrafast bootstrap (UFBoot; 1,000 replicates) with NNI optimization and SH-like approximate likelihood ratio tests (aLRT; 1,000 replicates) (parameters: -m MFP -bb 1000 -bnni -alrt 1000 -seed 42). The tree was re-rooted using archaeal MAGs as an outgroup and visualized in iTOL. (2) Bootstrap support ≥90 is denoted by gray circles on the corresponding branches. (3) Taxonomic affiliations are indicated by colored bands extending to the tree tips for each MAG. (4) Virus–host linkages are denoted by red stars, marking MAGs with evidence of infection by ≥1 virus. (5) The first ring shows the metabolic guilds (A–G) based on protein-content clustering. (6) The second ring shows the vegetation/sample type from which each MAG was reconstructed (green = mangrove, orange = seagrass, blue = *Sporobolus*, red = water). (7) The third ring indicates MAGs encoding dissimilatory sulfite/sulfur reductase (Dsr) genes associated with sulfur metabolism; pink squares, purple circles, and green stars represent Dsr reductive, Dsr oxidative, and Dsr reductive dismutation types, respectively, as detected by DiSCo. (8) The outer bar plot (light purple) depicts the relative abundance of each MAG across samples.

MI sediments from three different vegetation types (Fig. 1) were sampled in fall and summer (to capture seasonality, with trips split into day and night sampling). Sediment cores and overlying water were collected from mangrove, *Sporobolus*, and submerged seagrass. DNA was extracted from overlying water and sediment cores meant to primarily target depths of 2, 12, and 20 cm (16, 17, and 16 samples, respectively), with sparsely sampled depths at 40, 48, 56, and 58 cm (one sample each) and two samples from overlying water. For downstream analysis, sediment samples were grouped into three depth layers: top (2 cm), middle (12 cm), and bottom (20, 40, 48, 56, and 58 cm). This resulted in a total of 55 samples analyzed in this study. Assembly and binning of all these samples resulted in a total of 491 MAGs (>50% complete and <10% contamination). Of these MAGs, 472 were classified as bacteria and 19 as archaea, comprising 28 bacterial and 4 archaeal phyla according to the Genome Taxonomy Database (GTDB) (Release 220, Fig. 2; Table S2). Archaeal (super)phyla consisted of Asgardarchaeota (number of MAGs *n* = 3), Methanobacteriota_B (*n* = 1), Nanoarchaeota (*n* = 1), and Thermoproteota (*n* = 14) (Table S2), while the bacteria consisted mainly of Desulfobacterota (*n* = 180), Pseudomonadota (*n* = 142), and Chloroflexota (*n* = 64) (Table S2). We estimated the relative abundance of these phyla based on mapped reads to all the MAGs (see Materials and Methods), and from this, Desulfobacterota was the most abundant phylum, followed by Pseudomonadota and Chloroflexota (Fig. 2; Fig. S1A). To assess the diversity and structure of the 491 bacterial and archaeal MAGs, phylogenetic analysis was performed based on concatenated alignments of 37 conserved ribosomal proteins (RP37) of all MAGs. The GTDB-Tk classification reported members of 25 phylum-level lineages in addition to the dominant Desulfobacterota, Pseudomonadota, and Chloroflexota (Fig. 2). In the archaeal group, Thermoproteota was the most prominent phylum.

**Fig 3:**
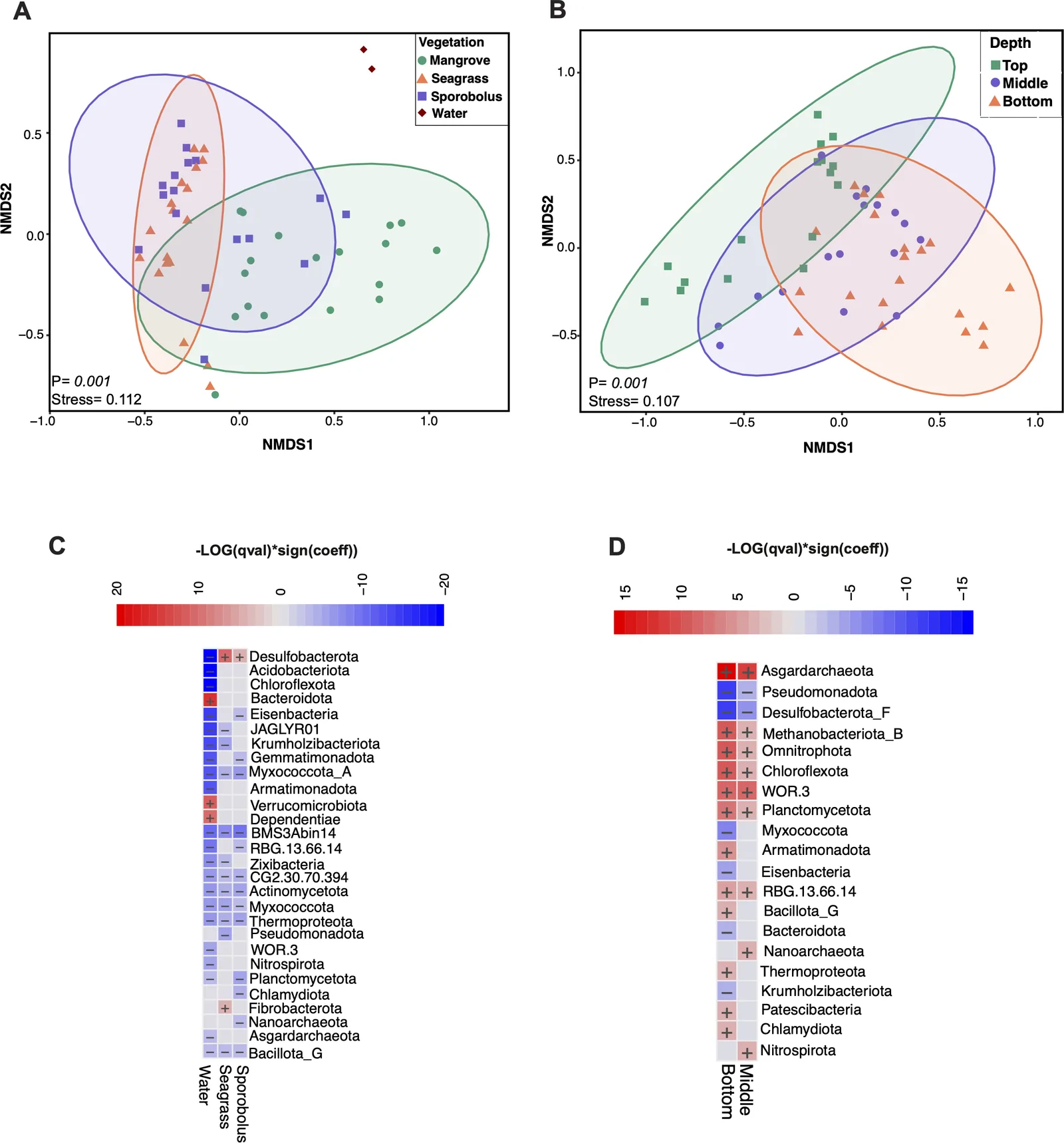
Significant associations of microbial phyla to specific vegetation types and sediment depths. (**A**) NMDS plot of Bray–Curtis distances showing clusters of 55 metagenomic samples with similar microbial communities across vegetation. (**B**) NMDS ordination of microbial communities based on Bray–Curtis distances, showing sample clusters at top (2 cm), middle (12 cm), and bottom layers (20, 40, 48, 56, and 58 cm) of sediment depths. PERMANOVA was performed to assess whether microbial community composition differed significantly (*P* < 0.05) across vegetation types and depth levels of sediment cores in cm depths (Table S2). (**C**) The heatmap gives a summary of all microbial communities with significant associations to certain vegetation types detected by MaAsLin 2’s linear model. Taxa that have significant associations to certain vegetation types (*Sporobolus*, seagrass, and water) are colored (red or blue) and overlaid with an addition (+) or subtraction (−) sign to indicate positive or negative compared with the reference group (mangrove), respectively; i.e., all effects are interpreted relative to mangrove vegetation. (**D**) Microbial taxa associated with specific depth. Heatmap legend color: −log(*q*-value) × sign (coefficient) obtained from MaAsLin 2’s linear model. Red color with plus sign overlay indicates a strong association to the reference depth; top layer (2 cm). The blue color (−) reveals a negative association to the top layer (2 cm depth). Empty cells indicate no significant association.

To assess differences in microbial community composition based on vegetation types, we performed a non-metric multidimensional scaling (NMDS; using Bray–Curtis distance) analysis based on the relative abundances of the MAGs. NMDS analysis shows that microbial communities associated with mangrove are different from those in seagrass and *Sporobolus* based on their abundance profiles. However, seagrass and *Sporobolus* share similar compositions. As expected, the overlying water samples have distinct microbial composition, forming separate clusters from the sediments. To further confirm that vegetation was an important factor affecting microbial presence and abundance, we performed PERMANOVA statistical analysis, which revealed that microbial communities differ by vegetation types (*P* < 0.05, *R*² = 0.32018, stress = 0.112; Fig. 3A; Fig. S2). We also found that microbial communities vary significantly across different sediment depths (*P* < 0.05, *R*² = 0.18043, stress = 0.10711; Fig. 3B; Fig. S3).

To identify phyla associations with vegetation types or water, we performed MaAsLin2 linear modeling (34, 35) based on MAG relative abundance profiles. Several positive and negative associations of microbial phyla with vegetation or sediment depth were identified (Fig. 3C and D; Fig. S2 and 3). The statistical significance indicates that each phylum was significantly different from the reference group (Table S2; see Materials and Methods).

Focusing on the three most prominent bacterial phyla based on our data set, we found that Desulfobacterota was more enriched in *Sporobolus* and seagrass than in mangrove sediment (reference group; see Materials and Methods) (Fig. S2A through C). Chloroflexota had no significant association with any vegetation type and was less abundant in the overlying water samples relative to mangrove (Fig. S2D). Pseudomonadota (formerly known as Proteobacteria) was significantly depleted in seagrass compared to mangrove (Fig. S2E). However, in *Sporobolus* and overlying water, no significant association was observed (Fig. S2E) in comparison to mangrove. Interestingly, MaAsLin2’s linear model on the sediment depth (using top layer as reference group) of the archaeal groups revealed high enrichment of Asgardarchaeota and Methanobacteriota_B in the middle and bottom layers compared to the top sediment layer (Fig. S3A through D), likely due to anoxic conditions typical of mangrove sediments beyond the first few millimeters, which are favorable for obligate anaerobes (36).

Nanoarchaeota and Thermoproteota were also abundant in the middle and bottom layers of the sediments, respectively (Fig. S3E and F). Some bacterial phyla were also enriched according to the sediment depth (Fig. S3). Notably, Pseudomonadota and Desulfobacterota_F were more enriched in the top layer (reference group) of the sediment than the rest layer (middle and bottom) (Fig. S3G through J). In contrast, Chloroflexota was more abundant at the middle and bottom layers than at the top layer of this sediment (Fig. S3K and L). Taken together, these phyla show distinct associations with vegetation type and depth layer (Fig. 3).

### Metabolic profiling of microbes via protein clustering

To characterize the functional capacities of these sediment microbes, we evaluated the presence of protein families involved in major nutrient cycles, and we found that most MAGs in this study showed a high prevalence of genes connected to sulfur, nitrogen, and iron metabolism (Fig. S4A through D; Table S3). Next, we defined groups of MAGs with shared metabolic traits (hereafter referred to as metabolic guilds) as previously described in (37–39) (Fig. 2). In brief, we clustered the 491 MAGs (Fig. 2) into seven metabolic guilds (guilds A–G) based on their predicted protein content.

Guilds A and B (deep and light brown colors in Fig. 2) consist of 94 and 81 MAGs, respectively, and both represent exclusively Desulfobacterota. Guild A is dominated by class Desulfobacteria with only 2 MAGs belonging to class DSM-4660. On the other hand, guild B consists of classes Desulfobacteria (49 MAGs), Syntrophobacteria (23 MAGs), DSM-4660 (7 MAGs), and Desulfarculia (2 MAGs). Based on the counts of MAGs per guild, we found notable differences in the vegetation types from which MAGs in each guild were recovered. In guild A, nearly 40% of Desulfobacterota MAGs were recovered from seagrass sediments, whereas in guild B, the largest fraction of MAGs (45.7%) was obtained from *Sporobolus* sediments. This pattern suggests differences in ecological preferences between the distinct metabolic guilds in Desulfobacterota (Fig. S5A). Given the distinct variation in protein content within Desulfobacterota, we analyzed differences in genome size between metabolic guilds A and B. Since a Shapiro–Wilk test indicated non-normal distribution (*W* = 0.98, *P* = 0.02), we compared guilds A and B using the Wilcoxon rank-sum test. Our analysis indicated that guild B had larger genome sizes (median 4.2 vs. 3.2 Mbp; Wilcoxon *W* = 5942, *P*-value = 1.639e-10) than guild A (Fig. S5B). This difference in Desulfobacterota agrees with our previous observations within this phylum (38). Although both guilds encode dissimilatory sulfite reductases (Dsr), their genetic repertoires differ in the types of Dsr. All MAGs in guild A encode reductive *dsr* genes, while about half encode genes for Dsr dismutation, with no evidence of oxidative *dsr* genes. In contrast, most of the MAGs in guild B encode *dsr* reductive and dismutation genes, with oxidative *dsr* genes being the least observed in this metabolic guild. MAGs in these A and B metabolic guilds are also among the most abundant MAGs recovered in this study (see Materials and Methods) (40) (Fig. 2; Table S2; Fig. S1A; Table S3).

Of the 142 MAGs from Pseudomonadota, 140 MAGs were assigned to metabolic guilds C and D. Metabolic guild C (dark cyan in Fig. 2) consists of 125 MAGs from class Gammaproteobacteria (*n* = 121) and a few Alphaproteobacteria (*n* = 4). This guild includes more MAGs derived from mangrove (60.8%) than other vegetation types. In contrast, metabolic guild D (light yellow, Fig. 2) consists of 15 MAGs, all of which belong to the class Alphaproteobacteria (order Rhizobiales) and harbor genes involved in nitrogen cycling, a characteristic feature of this order (41, 42). MAGs in this guild were found in mangroves (40.0%) more often than any other vegetation type. A small fraction of MAGs in guild C (3.2%) were associated with water samples, whereas no MAGs from guild D were found in water (Fig. S5A). Within metabolic guild C, only 69 MAGs (49.3%) encode genes for *dsr*-based oxidation (Table S4). However, *dsr* genes were absent in guild D. Based on the relative abundance profile of the MAGs by CoverM (40), guilds C and D (Pseudomonadota) together represent the second most abundant group from this data set (Table S2; Fig. S1A).

Metabolic guild E (light cyan color in Fig. 2) consists of 101 MAGs belonging to different taxonomic categories (see Table S3). Most of the MAGs (63%) were found in mangroves, while 17%, 12%, and 8% were found in *Sporobolus*, seagrass sediments, and water, respectively. Within this guild, 27 MAGs carry reductive-type dsr genes, with Gemmatimonadota (9 MAGs), Acidobacteriota (7 MAGs), and JAGLYR01 (3 MAGs) contributing the largest numbers of reductive Dsr (see Table S4). 14 MAGs possess genes for oxidative Dsr, and seven MAGs contain genes for reductive dismutation. Although Chloroflexota is primarily represented in a distinct guild (guild F, described below), seven MAGs were assigned to guild E. These MAGs belonged to classes Dehalococcoidia (5 MAGs) and Limnocylindria (2 MAGs). In contrast to the other Chloroflexota MAGs, which typically range from 3 to 5 Mb, these classes have relatively small genome sizes (0.7–1.7 Mb). Their differences in metabolic activity and genome size likely reflect class-level distinctions. Within Dehalococcoidia, 4 out of 5 MAGs were associated with mangrove sediments, while Limnocylindria were all found in seagrass sediments. Overall, the relative abundances of MAGs in this guild are lower than those observed for other MAGs (Fig. S1A).

We found 57 MAGs in guild F (teal in Fig. 2), primarily from Chloroflexota (class Anaerolineae) (see Table S3). Of the guild F representatives, 59.6% of them were recovered from mangrove sediments (Fig. S5A). This is consistent with previous studies describing elevated abundances of Chloroflexota in sediments from mangrove forests near the coast of pPeninsular Malaysia (43). 16 MAGs belonging to class Anaerolineae in this guild encode reductive sulfur pathways, specifically reductive Dsr, converting sulfate or sulfite into reduced forms of sulfur, while only 1 MAG encodes oxidative Dsr (Table S4), highlighting a specialized role in sulfur metabolism. Based on the MAG-level relative abundances, Chloroflexota is among the top most abundant phyla in this dataset, together with Desulfobacterota and Pseudomonadota (Table S2, Fig. S1A).

The archaeal group consists of 18 MAGs that fall largely under metabolic guild G (Fig. 1; light orange), with the exception of MAG belonging to the Nanoarchaeota that was assigned to guild E. A total of 72.2% of MAGs in this guild were recovered from mangrove sediments (Fig. S5A). We found no evidence of genes encoding any of the listed Dsr in guild G (see Fig. 2). The relative abundance of MAGs within the archaeal group is lower than bacterial MAGs (Fig. 1; Table S2, Fig. S1A).

Iron is an important micronutrient (44), and its function in microbes is connected to processes such as sulfur, nitrogen, and carbon cycling through oxidative and reductive reactions (45). Given that most MAGs in this study showed high potential for iron cycling based on entropy scores (46) (Fig. S4D), we explored the presence of marker genes associated with iron-related processes across each metabolic guild. We identified genes related to iron storage, gene regulation, and transport and acquisition mechanisms, including siderophores. Notably, guild A showed the highest iron storage and general iron acquisition/transport, and guilds E and F showed a particularly high iron gene regulatory genes, with guild E showing the highest number of these genes. Guild C showed the highest iron acquisition siderophore transport potential, while siderophore transport genes were more prominent in guilds B, C, D and E. These observations suggest that the MAGs associated with these guilds play roles in iron cycling in MI sediments.

To examine roles in carbon cycling, we screened for methyl coenzyme M reductase (MCR), the key enzyme responsible for the final steps of methanogenesis and the first step of anaerobic respiration of methane in anaerobic methanotrophic archaea (ANME) (47). MCRs were not identified in either assemblies or MAGs using our previously compiled MCR database (48, 49). This absence may be due to one or more of the following reasons: (i) bias in archaeal recovery during the DNA extraction, (ii) methanogens being constrained to deeper layers beyond the 2-40 cm core depth sampled here, or (iii) insufficient sequencing depth to detect low abundance taxa.

### Viral–host interaction in MI coastal sediments

To characterize the viruses in MI sediments, we searched for viral genomes across all 55 metagenomic samples and assessed their potential influence on microbial communities (Fig. 2). We identified 13,962 viral scaffolds from the assemblies. Of these, 6,366 scaffolds met vRhyme (50) quality criteria for binning; these were binned to obtain 1,451 vMAGs. After removing redundant bins and retaining only vMAGs with at least 10 scaffolds to meet the required quality threshold, 1,097 vMAGs remained, comprising 3,486 scaffolds (see Materials and Methods; Fig. S7A). In addition, the 354 bins that did not meet the quality threshold were deconstructed into individual scaffolds, from which we retained 2,880 viral scaffolds as uncultivated viral genomes (UViGs) for downstream analyses. Among the vMAGs, five were classified as complete, 56 as high quality, 163 as medium quality, and 834 as low quality. Two UViGs were high quality, 16 were medium quality, and 2,539 were low quality (Fig. S7B and D).

Most viral genomes displayed a lytic cycle, including 1,049 vMAGs and 2,848 UViGs, whereas a small fraction (48 vMAGs and 32 UViGs) were lysogenic (Fig. S7C and 5E; Table S5) according to VIBRANT. Genome size varied widely; the vMAGs ranged from 7.6 to 261.6 kb, while UViGs spanned 3.0–150.0 kb (Fig. S7B and D; Table S5).

**Fig 4:**
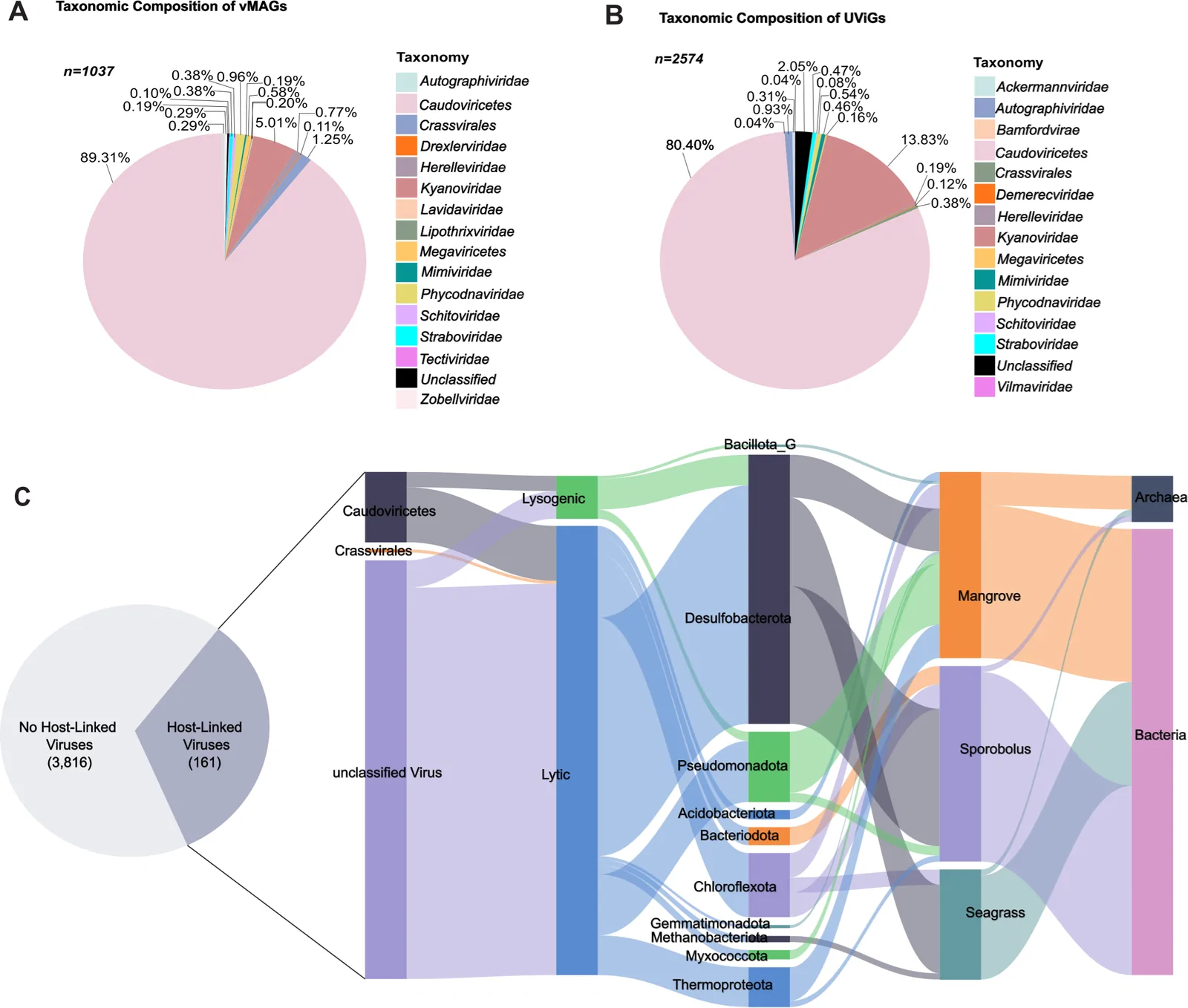
Taxonomic classification of viruses and their predicted host. (**A**) Pie chart represents the percentage distribution of viral classes in MI sediment vMAGs. (**B**) Pie chart representing the percentage distribution of viral classes in MI sediment UViGs. “Unclassified” here represents viral genomes with no taxonomic assignment based on geNomad. (**C**) Pie chart (left) shows the number of viral genomes with a predicted host taxonomy based on iPHoP. Alluvial plot (right) shows the taxonomic profile of the host-associated viruses, their replication cycle separated into lysogenic and lytic, their host phyla, their associated vegetation types, and host domain.

Taxonomic assignment was inferred from viral gene content using conserved marker proteins and genome-wide similarity to reference viral lineages (see Materials and Methods). Using this approach, taxonomy was assigned to 90.8% of viral genomes (3,611 of 3,977) with most identified as double-stranded DNA (dsDNA) genomes. Most of the assigned viral genomes were classified into the realm of *Duplodnaviria* (97%), with smaller percentages assigned to *Varidnaviria* (1.4%) and *Adnaviria* (0.03%). Within *Duplodnaviria*, the majority belonged to the class *Caudoviricetes*, and a subset were further resolved into the orders *Kyanoviridae*, *Crassvirales*, *Zobellviridae*, *Autographiviridae*, *Schitoviridae*, *Herelleviridae*, *Straboviridae*, *Drexlerviridae*, *Demerecviridae*, and *Ackermannviridae* (Fig. 4A and B; Table S5).

Viruses classified within *Varidnaviria* belonged to the class *Megaviricetes*, whereas *Adnaviria* virus was placed in the class *Tokiviricetes*. A small percentage of the viral genomes (55 of 3,611; 1.5%) were labeled as “Unclassified” by geNomad (51) and could not be confidently assigned to an existing taxonomic group. Overall, these viral genomes were mostly of a lytic cycle (3,897 of 3,977; 98%). The high proportion of observed lytic viruses in these sediments might indicate more nutrient availability.

We predicted viral hosts using iPHoP (52), which assigned hosts to ~4% (*n* = 161) of the 3,977 viral genomes (vMAGs and UViGs) (see Materials and Methods). Many of the predicted hosts belonged to Desulfobacterota (*n* = 88, metabolic guilds A and B). The viruses infecting this host are predicted to have a lytic lifestyle, and the majority of these have unknown viral taxonomic classification. The unclassified viruses infecting Desulfobacterota are mostly associated with the *Sporobolus* and seagrass vegetation, while those associated with Pseudomonadota are mostly present in mangrove, and other taxa are sparsely distributed in different vegetation types (Fig. 4C). Other predicted hosts included Pseudomonadota (*n* = 23), Chloroflexota (*n* = 21), and Thermoproteota (*n* = 13), with a few other viruses being associated with Bacteroidota (*n* = 6), Acidobacteriota (*n* = 3), Myxococcota (*n* = 3), Methanobacteriota (*n* = 2), Gemmatimonadota (*n* = 1), and Bacillota_G (*n* = 1), as shown in Fig. 2.

Based on metabolic guilds, we found no virus to host linkages in MAGs found in guild D, while viruses to host linkages were identified in guild C. All viruses associated with metabolic guild C are predicted to have a lytic cycle, with the majority lacking viral taxonomic identity (Table S5). Similarly, MAGs in metabolic guilds E (particularly Limnocylindira), F, and G, have lytic cycles and lack viral taxonomic identity. Viruses linked to host MAGs in this metabolic guild F were mostly observed in the phylum Thermoproteota and exhibited lytic cycles with no taxonomic information (Fig. 2; Table S5).

The majority of the host-linked viruses (91%, 147/161) are predicted to have a lytic cycle, yet most of them could not be assigned a taxonomic identity using geNomad (51). Those that could be classified were mostly putatively lytic members of the *Caudoviricetes* (11%, 18/161) that appear to primarily infect Chloroflexota (Fig. 4C). The unclassified viruses (79.5%, 128/161) were predicted to infect Desulfobacterota, Pseudomonadota, Thermoproteota, Gemmatimonadota, Methanobacteriota, and Myxococcota. The unclassified viruses infecting Desulfobacterota are mostly associated with the *Sporobolus* and seagrass vegetation, while those associated with Pseudomonadota are mostly present in mangrove, and other taxa are sparsely distributed in different vegetation types (Fig. 4C).

### Carbohydrate-active enzyme genes in MI MAGs

Carbon turnover in marine sediments (53) and coastal ecosystems (54) is mediated by microbial communities. To assess the capacity of MAGs for organic matter degradation in the MI coastal ecosystem, we searched for carbohydrate-active enzymes (CAZymes) (see Materials and Methods). We identified different classes of CAZymes involved in the synthesis, modification, and degradation of organic matter (Fig. S8A). These classes are auxiliary activities (AAs), carbohydrate binding modules (CBMs), carbohydrate esterases (CEs), glycoside hydrolases (GHs), glycosyltransferases (GTs), and polysaccharide lyases (PLs) (Table S6). In total, 199 CAZyme families were found in these microbial MAGs, of which the most were glycosyltransferases (GT4, GT2, and GT51), carbohydrate-binding modules (CBM48), and glycoside hydrolases (GH13, GH3, GH5, GH23, GH35, and GH57) (Fig. S8A). To further assess the functional roles of these enzymes, we examined their predicted cellular localization (55). Approximately 60% of CAZymes were predicted to be localized in the cytoplasm, while about 16% were of unknown location. The remaining 24% were distributed across the cytoplasmic membrane, cell wall, extracellular, outer membrane, and periplasmic space (Fig. S18B, Table S6), suggesting both intracellular carbohydrate metabolism and extracellular or surface-associated organic matter degradation.

Distinct taxonomic and habitat-specific patterns were observed in CAZyme composition. Glycosyltransferase genes, particularly GT2 (cell wall and membrane polysaccharide synthesis) and GT4 (lipopolysaccharide synthesis and sugar transfer), were observed in most phyla, especially genomes assigned to metabolic guild E, but were comparatively rare in Desulfobacterota, Pseudomonadota, and Chloroflexota (metabolic guilds A, B, C, and F), which encoded few or no GT2/GT4 genes. Mangrove-associated MAGs exhibited the highest abundance of CAZyme genes, with families GT2, GT4, and GH13 (involved in complex carbohydrate degradation) being significantly more prevalent in these MAGs compared to those from other vegetation-associated sediments (Fig. S8C).

### Viral implications in carbon cycling

We screened for auxiliary viral genes (AVGs) across both vMAGs and UViGs to understand how viruses at MI may impact the metabolisms of their microbial hosts using CheckAMG. A total of 1,269 AVGs were identified (see Materials and Methods) (Table S5). Four hundred nineteen AVGs lacked a KEGG pathway annotation (56); thus, we could not confidently assign function to the genes. However, we found AVGs with a broad range of functions, including those involved in genetic information processing, protein chaperones, carbohydrate and amino acid metabolism, DNA replication, viral replication, and other metabolic activities (Fig. S9A). Out of 1,269 detected AVGs, only 2.1% of these viral genes could be linked to our predicted host phyla (Fig. S9B; Fig. 4). From this, 0.2% of AVGs were linked to each of Acidobacteriota, Bacteroidota, Chloroflexota, Methanobacteriota_B, Myxococcota, Pseudomonadota, and Thermoproteota*,* while 1% were linked to Desulfobacterota (Fig. S9B). These AVGs are broadly distributed in the three vegetation types (mangrove, *Sporobolus*, seagrass) and overlying water samples. Functional classification of the AVG types across all vegetation revealed a high percentage of regulatory AVGs (57.4%) (Fig. S9C) and a smaller proportion of metabolic genes (35.5%). Lastly, physiological viral genes were the lowest observed AVGs from our data set (7% approximately) (Fig. S9C).

Given the high presence of viral MAGs with lytic replication strategies, we searched for viral CAZymes to identify viruses that may be actively involved in carbon metabolism. We identified multiple classes of CAZyme families, most notably GH19 (chitin degradation), GT25 (lipopolysaccharide core synthesis), GT11 (glycoprotein N-glycan branching), GT4 (glycolipid and polysaccharide biosynthesis), and GH24 (peptidoglycan hydrolysis) (Fig. S9D). Together, these viral CAZymes may allow viruses to modify the host environment in ways that optimize viral replication, which may contribute to high turnover of organic matter and nutrients in these sediments.

## DISCUSSION

**Fig 5:**
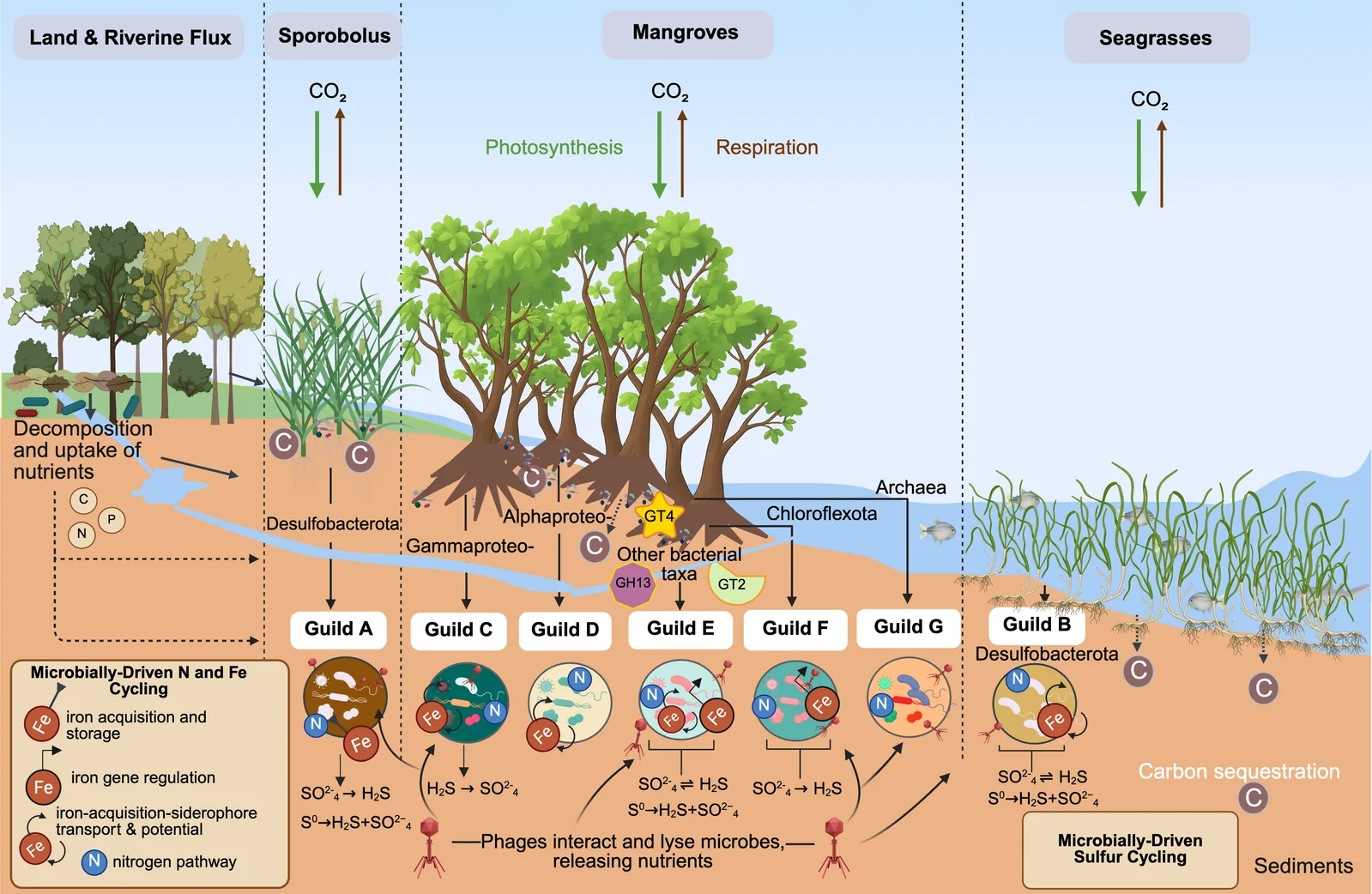
Vegetation-driven patterns of microbial diversity and biogeochemical cycling in coastal Texas wetlands. From left to right, the figure depicts upland flow of water into the three vegetation types (*Sporobolus*, mangroves, and seagrasses). Microbial communities facilitate nutrient decomposition and uptake within these vegetated sediments. Each vegetation type supports distinct metabolic guilds that mediate sulfur, iron, and nitrogen cycling, enhancing nutrient turnover and ecosystem stability. Metabolic guilds were assigned to the vegetation in which their relative distribution was highest. For instance, guilds A and B were placed in *Sporobolus* and seagrass sediments, respectively, because the number of MAGs belonging to this guild was highest in those vegetation types. Microbes within metabolic guild E show a high presence of CAZymes in mangrove sediments. Viruses interact with hosts in the guilds by infecting and lysing our host cells. Vertical and horizontal arrows indicate water and nutrient flow through these vegetation types, while carbon storage is facilitated by carbon dioxide sequestration and accumulation of organic carbon in sediments. Created with Biorender.com.

Here, we performed the first genome-resolved metagenomic study in the Mud Island of mangrove-cordgrass ecotones, which represent some of the northernmost distributions of *A. germinans*. By recovering many MAGs across spatial and temporal gradients, this study provides a comprehensive view of microbial diversity and functional potential in MI coastal sediments. The integration of taxonomic, metabolic, and viral analyses reveals how vegetation type structures microbial guilds and, in turn, shapes biogeochemical cycling in coastal wetlands (Fig. 5).

High inputs of organic carbon and nutrients from adjacent upland environments fuel anaerobic metabolisms in coastal wetland sediments, particularly sulfate reduction (57). In MI, anaerobic processes, especially sulfate reduction, appear to be primarily driven by members of guild A, dominated by Desulfobacteria, which encode genes involved in sulfate reduction as well as nitrate reduction and iron storage. A similar metabolic profile was observed in guild B, composed mainly of Desulfobacteria and Syntrophobacteria, in seagrass sediments, although these taxa exhibit a greater potential for iron acquisition via iron-siderophore transport. The dominance of Desulfobacterota across seagrass and *Sporobolus* sediments aligns with previous observations of their metabolic versatility and predominance in anaerobic coastal environments (58, 59). Differential abundance analyses further revealed strong associations between microbial taxa and vegetation types, as has been previously reported (60, 61). Notably, Desulfobacterota, Pseudomonadota, and Chloroflexota were more present and abundant in the summer months in MI vegetated sediments, consistent with seasonality shifts in salinity and associated biogeochemical conditions, including sulfate availability and iron cycling in northwestern Gulf of Mexico estuaries (62–64).

In mangrove sediment communities, we observed a greater diversity and abundance of bacteria compared to those of archaea, in accordance with previous reports which showed that bacteria species have broader niche breadths and higher alpha diversity than archaea and are more widely distributed across vegetation zones (65). MAGs in guild C (Gammaproteobacteria), associated with mangrove sediments, encode pathways for sulfur oxidation, nitrogen fixation, and dissimilatory nitrate reduction. This group could also participate in iron acquisition through siderophores in this environment, as they contain multiple ExbD-family proteins and TonB, together with FpvE-family permease and FeoAB-family ferrous iron transporters. In gram-negative bacteria, high-affinity ferric iron acquisition requires TonB-ExbB-ExbD complexes, so the presence of these complexes suggests that guild C MAGs have a high-affinity competition for iron in this organic-rich environment, highlighting their multifunctional roles in sulfur, nitrogen, and iron cycling, as also reported for mangrove sediment microbes (66, 67). Other mangrove-associated guilds further contribute to ecosystem functioning. Guild D (Alphaproteobacteria) encodes nitrogen reduction and siderophore-mediated iron acquisition pathways but lacks *dsr* genes indicating functional specialization within mangrove sediments. Guild E, dominated by Gemmatimonadota, contains genes predicted to be involved in both oxidative and reductive sulfur metabolism, along with iron regulatory functions, pointing to a role in redox balancing across sulfur and iron cycling. In contrast, genes for sulfate reduction were most prominent in Acidobacteriota, in accordance with a recent study of marine Acidobacteriota encoding canonical sulfate-reducing pathways, acting as important sulfate reducers (68). Chloroflexota, primarily within guild F, were broadly distributed in mangrove sediments, and many harbored genes encoding for sulfate reduction, including QmoC_SRO, anQmoB2_SRO, and a few DrsAB genes, as well as iron regulatory genes, suggesting their role in these processes. Together, these findings illustrate a tightly coupled sulfur–iron–nitrogen network distributed across multiple guilds, with emerging evidence that Gemmatimonadota, Acidobacteriota, and Chloroflexota contribute to sulfur and iron transformation in marine sediments (37, 68, 69), and indicate that mangrove sediments support particularly complex metabolic interactions.

The archaeal communities at MI were dominated by Thermoproteota and Asgardarchaeota (guild G). They do not encode *dsr*-mediated sulfur cycling genes, nor do they encode prominent iron cycling genes. Thermoproteota are widely distributed across different environments (70–72) and are among the most abundant archaeal groups in this study, similar to their dominance in the planktonic archaea communities of the Pearl River Estuary (71). Asgardarchaeota were more prevalent at deeper sediment depths (12 and 20 cm), consistent with previous studies (73, 74). Although sulfur metabolic genes were present in this guild, the pathway appears incomplete as *dsr* genes were not found in any members of the guild. While the absence of Dsr in guild G could be attributed to genome incompleteness, the possibility exists that metabolic handoffs among bacteria and archaea in these communities drive sulfur and carbon metabolism in conjunction with viral activity.

MI viruses were shown to infect dominant microbial metabolic guilds (A, B, C, and F) and may modulate host metabolic activity via AVGs, influencing sediment nutrient and carbon cycling in the process. Most predicted AVGs occurred in viruses inferred to infect guild B (Desulfobacterota) hosts, although at a relatively low (1%) proportion. Viral gene annotations suggest that MI sediment viruses encode putative AVGs that may modulate a variety of microbial metabolic pathways, including genetic information processing, carbohydrate and amino acid metabolism, and biogeochemical cycling. Similar roles for viral AVGs have been documented in sedimentary systems and hydrothermal vents (75, 76) supporting the idea that viruses enhance host metabolic flexibility in fluctuating environments. Most of our viral-encoded CAZymes were obtained from water samples, with dominant families including GH19, GH24, GT25, GT11, GT4, and GT6. Glycoside hydrolases GH19 and GH24 have been suggested to aid viral entry and cell wall degradation (77). Glycosyltransferases enable viruses to modify their proteins and DNA with sugars to evade host detection and inhibit host molecular functions (78). These viral CAZymes likely contribute to microbial turnover and nutrient release, further reinforcing the role of viruses in shaping carbon and nutrient dynamics in MI sediments.

### Conclusion

This study provides a comprehensive genomic assessment of microbial and viral interactions across sediments associated with diferent vegetation types in MI coastal estuarine ecosystem. Previous studies in this environment have primarily focused on nutrient dynamics, hydrology, and water quality, and planktonic or faunal responses (79–84), while sediment microbial and viral communities and their links to vegetation have remained largely unexplored. Here, we provide an extensive characterization of microbial community structure and functional potential across vegetation types and sediment depths, demonstrating that both are strongly structured by vegetation, depth, and environmental gradients. Our study shows that mangrove, *Sporobolus*, and seagrass sediments harbour functionally distinct microbial communities, with each habitat characterized by specific metabolic guilds involved in sulfur, nitrogen, iron, and carbon cycling. Viruses preferentially infect dominant microbes within each vegetation type, suggesting that they play an important role in regulating microbial populations and influencing nutrient and carbon turnover across the ecosystem. The coexistence of metabolic guilds mediating sulfur, nitrogen, iron, and carbon transformations further highlights the central role of microorganisms in sustaining biogeochemical processes in these highly productive ecosystems. Together, these findings provide a genomic framework for understanding ecosystem functioning at the land–sea interface and underscore the importance of vegetation-driven microbial and viral proccesses in shaping coastal carbon and nutrient cycling.

## MATERIALS AND METHODS

### Sampling

Mud Island sediments were sampled during three field campaigns in fall (November 2017) and summer (June and August 2018), during both day and night across three vegetation types (submerged seagrass, *Sporobolus*, and black mangrove). One independent sediment core was collected for each vegetation type during each day/night sampling event, resulting in a total of 18 cores. Sediment cores were subsectioned into defined depth horizons, primarily targeting: 2–4 cm, 12–14 cm, and 20–22 cm, with additional deeper horizons sampled more sparsely (40–42 cm, 48–50 cm, 56–58 cm, and 58–60 cm). The resulting data set comprised 55 metagenomic samples distributed across vegetation types, sediment depths, seasonal campaigns, and diel conditions (Table S1). Cores were collected in 70 cm polycarbonate tubes. Recovered cores were frozen upright and stored at −80°C until processing. Sediment profiles displayed a top ~15 cm of vegetated, porous sediment, with a stark shift to reductive sand containing iron sulfides.

### Metagenome sequencing and assembly

Sediment cores were stored at −80°C and sub-sectioned in 2 cm increments targeting 2, 12, and 20 cm for molecular analyses. DNA was extracted alongside RNA with a QIAGEN RNeasy Kit with DNA elution accessory kit (QIAGEN, Germantown, MD, USA). For each extraction, we aimed to obtain a total of 100 ng of DNA, which required 2–9 replicate extractions of 5 g of sediment each, depending on yield. An extraction blank was carried throughout as a control for contamination to test for contaminants both within the lab and within the extraction kits. The Zymo Clean and Concentrator kit was used to clean and concentrate extractions (Zymo Research, Irvine, CA, USA). Quality and concentration checks were performed using PCR, Biospectrometer (Eppendorf), and a Qubit 3.0 fluorometer (Thermo Fisher Scientific, Singapore), respectively. Extracted DNA was sent to the Department of Energy Joint Genome Institute (JGI) for Illumina Nextera library preparation and 2 × 150 bp sequencing (Illumina, San Diego, CA, USA). Quality control and assembly were performed per IMG v5.0 pipeline standards (85), with adapter trimming, quality control, and contaminant removal using BBTools v38.59 (86) rqc.filter2.sh script (parameters: rna = f trimfragadapter = t qtrim = r trimq = 0 maxns = 3 maq = 3 minlen = 51 mlf = 0.33 phix = t removehuman = t removedog = t removecat = t removemouse = t khist = t removemicrobes = t sketch kapa = t clumpify = t tmpdir = null barcodefilter = f trimpolyg = 5). Assemblies were performed with metaSPAdes v3.13.0 (87).

### Genomic binning

Genomic reads from MI sediment samples (*n* = 55) were mapped to a combined FASTA of all individual metaSPAdes assemblies with BWA-MEM v0.7.17-r1188 (88) and Samtools v1.10 (89) using the bwa_bam_map.sh script: https://github.com/valdeanda/Useful_scripts/blob/master/bwa_bam_map.sh. BAM integrity and sorting were, respectively, assessed with samtools quickcheck and samtools stats using the bam_check.sh script: https://github.com/valdeanda/Useful_scripts/blob/master/bam_check.sh. A depth file was created from indexed and coordinate-sorted BAMs with JGI_summarize_bam_contig_depths. Genomic binning was performed with MetaBat2 v2.15 (90) (parameters: –seed 4 -v -d –minCVSum 0 –saveCls –minCV 0.1 –minContig 3000), CONCOCT v1.1.0 (91) (parameters: –clusters 400 –kmer_length 4 –length_threshold 3000), MaxBin2 v2.2.7 (92) (parameters: -min_contig_length 3000 -prob_threshold 0.9 -markerset 40), and VAMB v3.0.0 (93) (parameters: -m 3000 -p 50 -e 500 --minfasta 100000; windowsize = 200, min cluster size = 1). For VAMB binning, the latent representation was trained for 500 epochs, after which the contiguous genome sequences, i.e., contigs, were re-clustered using bins ≥ 100 kilobases (kb) as medoids. Consensus MAGs were obtained with DASTool v1.1.2 (94) (parameters: --search-engine diamond). Completeness and contamination were estimated with CheckM v1.1.2 (95), with MAGs >50% complete and ≤ 10% contamination considered for further analysis. MAGs with high completeness and contamination >15% were manually refined with mmgenome v2.1.2 (96) by removing outlier contigs based on coverage values (https://github.com/imrambo/mmgenome2_workflow). Refined bins were re-evaluated with CheckM v1.1.2 and considered for analysis if the previous completeness and contamination criteria were met.

### MAG taxonomic classification and phylogenetic analysis

Initial taxonomic placement of MAGs into bacteria and archaea was performed with GTDB-Tk v2.3.2 (97) using the reference214 database. Phylosift v1.0.1 (33) was used to identify and align 37 conserved ribosomal proteins (RP) marker genes from each MAG (--search, –align –besthit). The resulting concatenated alignments of 37 conserved ribosomal proteins (RP) were merged into a single alignment, which was then trimmed with Clipkit v2.6.1 (m kpi-gappy -g 0.5), to remove poorly aligned and gappy sites (98). Maximum-likelihood phylogenies were created with IQTree v2.1.4 (99) using Model Finder Plus (100) with an SH-like approximate likelihood ratio test (101) and optimization of UFBoot trees by nearest-neighbor interchange (parameters: -m MFP -bb 1000 -bnni -alrt 1000 -seed 42). Then, the tree file was shown and annotated using iTOL v7 (102).

### MAG gene annotation and metabolic predictions

MEBS v1.2 (RRID:SCR_015708) (46) was used to determine MAG metabolic potential and score for carbon, sulfur, nitrogen, and oxygen metabolism. Hierarchical clustering of MAGs based on metabolic pathways was performed with the Seaborn clustermap function (euclidean distance, UPGMA/average clustering) via the mebs_vis.py script in MEBS v1.0. Iron-related genes were identified using FeGenie (103) to predict genes and gene neighborhoods related to iron acquisition, storage, and oxidation/reduction in bacteria and archaea. Prodigal v2.6.3 (104) was used to predict the genes of all 491 MAGs, and the output was further annotated using KofamScan v1.3.0 (105). DiSCo was used to screen all 491 MAGs for the presence or absence of dissimilatory sulfite reductase (Dsr) genes (106). It operates via Perl v3 framework and employs HMMER3 with default parameters set for Dsr identification. Carbohydrate-active enzymes (CAZymes) in MAGs were identified using the run_dbcan standalone pipeline v4.1.4 of dbCAN3 (107) (parameters: run_dbcan prok --out_dir). The genes encoding carbohydrate-degrading enzymes as described in the CAZymes database were identified by only retaining hits detected by at least two different tools. The subcellular localization of the selected CAZymes was determined using the web display mode of PSORTb v3.0 (55).

### Protein clustering

To understand protein similarity, we used MEBS (perl mebs.pl -input <genomes> -type genomic -comp option) to cluster proteins of all bacterial MAGs (*n* = 491) based on the presence and absence of protein domain families against the Pfam v34 database. Using the output of “mebs.pl,” we hierarchically clustered all the MAGs with the mebs_clust.py, applying the Jaccard distance and Ward variance minimization. We tested various maximum distance thresholds from 0.50 to 0.30 by 0.05, which produced five to 16 clusters. We chose 0.45 because the six protein clusters (guilds, labeled A–F) aligned well with the taxonomic profile of the MAGs.

### Relative abundance and ecological analysis

We used CoverM v0.70 (https://github.com/wwood/CoverM) pipeline to calculate read coverage for both MAGs and viral MAGs (vMAGs and UViGs). Abundance profiles were generated using the *coverm genome* subcommand with default alignment parameters (coverm genome --interleaved <fastq file> -d < genome dir> --methods count relative_abundance --min-covered-fraction 0 -t 60 -o < output_file.tsv>), using the 55 interleaved paired-end FASTQ reads to calculate the coverage metrics. Relative abundances of MAGs were subsequently summed within each bacterial/archaeal phylum, and relative abundances of vMAGs were summed within each viral class to generate a phylum/class-by-sample abundance matrix. Hierarchical clustering of phyla and viral class was then performed on Bray–Curtis dissimilarities calculated from this abundance matrix in R v4.1.2 (vegan), and the results were visualized as a clustered heatmap (pheatmap). All statistical analyses related to beta diversity were performed in R v4.1.2. Beta diversity of sediment microbial communities obtained from each vegetation type and depth was calculated using Bray–Curtis dissimilarities based on the relative abundances of microbial MAGs within each sample. Non-metric multidimensional scaling (NMDS) ordination scores were computed from the Bray–Curtis distances, and permutation multivariate analysis of variance (PERMANOVA) was used to assess the statistical significance of microbial community differences by vegetation type and depth level. MaAslin2 linear modeling (34) was performed on the relative abundance of all microbial MAGs to determine the multivariate associations. In the MaAslin2 models, mangrove (fixed effect) was specific as the reference group, so each significant association indicates that the abundance of a given phylum in *Sporobolus*, seagrass, or water differs significantly from its abundance in mangroves. The other testing associations were with sediment depth using top layer (2 cm) as the reference group (fixed effect). The statistical significance indicates that each phylum was significantly different from the reference group.

### Viral genome identification and binning

VIBRANT v1.2.1 (108) was used to screen for candidate viruses using default parameters on all the metagenomic assemblies from the 55 metagenomic samples; of 578,729 scaffolds that were read in as input, 435,765 scaffolds met minimum requirements with at least 1,000 bp and four ORFs. This analysis resulted in a total of 13,962 putative viruses, which were categorized as either lysogenic or lytic by VIBRANT based on the presence or absence of integrase or prophage marker genes, with lytic viruses showing an absence of these genes. The candidate viruses were binned using vRhyme v1.1.0 (50) on each of the 55 samples using the default parameters with the previously generated bam files. Altogether, we reconstructed 1,451 vMAGs. These vMAGs were further screened for high protein redundancy, where bins with ≥2 redundant proteins were broken down back into their individual viral scaffolds and retained in this data set as UViGs for downstream analysis. Similarly, vMAGs with ≥10 scaffolds were also broken down and added to the UViGs data set. The best vMAGs were linked with 1,500 Ns using the vRhyme script link_bin_sequences.py with default parameters. The N-linkers were important for downstream analysis such as CheckV and iPHoP, which require viral genomes to be on one scaffold as described below. vMAGs that had lysogenic and lytic viral scaffolds binned into one genome were assigned as lysogenic. SeqKit v2.9.0 was used to determine the size of the viral genomes, for both vMAGs and UViGs without N-links (109).

### Viral quality, taxonomy, and auxiliary viral genes identification

Viral genomes, including both binned and unbinned genomes (vMAGs with N-linkers and UViGs), were further screened for quality using CheckV1.0.1 (110) end_to_end pipeline with default parameters to identify complete, low-, medium-, and high-quality viral genomes. Taxonomic placement of viral genomes was performed using geNomad v1.8.1 (51), which employs a marker-based approach to assign viral sequences to specific taxa defined by the International Committee on the Taxonomy of Viruses (ICTV) (111). The “annotate” pipeline was run with default parameters. For CheckV and geNomad, vMAGs with 1,500 N-linkers and UViGs were used as inputs. To identify the presence of AVGs, CheckAMG v0.3.3 was run on vMAGs and UViGs using the default parameters. Genes with KEGG annotations were classified as “metabolic,” “physiological,” and “regulatory.” While many AVGs could be assigned to metabolic categories by their KEGG annotations, some AVGs showed “no hits” to any pathway description on the KEGG database. These viral proteins were still considered as AVGs, as they were predicted by other Profile HMM databases integrated into CheckAMG. Lastly, manual curation was performed to retrieve the pathway description of the AVGs from the KEGG database (https://github.com/AnantharamanLab/CheckAMG). Then, the files were imported into Rv4.12 for statistical analysis.

### Prediction of host–virus pairing

Virus–host pairings were predicted to understand the interactions of microbial communities in this coastal ecosystem using iPHoP v1.3.3 (https://bitbucket.org/srouxjgi/iphop). A custom database of 496 MAGs was added to iPHoP’s host database “Aug_2023_pub_rw.” Prior to this, the custom database was created using the “add_to_db” function (gtdbtk de_novo_wf --genome_dir --bacteria --outgroup_taxon p__Chloroflexota --out_dir; gtdbtk de_novo_wf --genome_dir --archaea --outgroup_taxon p__Altiarchaeota --out_dir) (97). Then, iPHoP “predict” command was performed on the viral genomes from vMAGs and UViGs with the following parameters: iphop predict --num_threads --fa_file --db_dir --out_dir to determine the host–viral linkages. The virus-host linkage with the highest confidence score was selected as the best predicted association. The inputs were vMAGs with 1,500 N-linkers and UViGs to enable a host to viral prediction per vMAG.

### Code availability

All scripts used for the analysis of the MI data set can be accessed from the following GitHub repository: https://github.com/ginafav/MANERR_project/tree/main.

## Data Availability

Data and annotations are available through JGI IMG as JGI Proposal ID 503452: https://genome.jgi.doe.gov/portal/Dynofmecosystem/Dynofmecosystem.info.html.

## SUPPLEMENTAL MATERIAL

Supplemental Figures msystems.01071-26-s0001.pdf Figures S1-S9.

Table S1 msystems.01071-26-s0002.xlsx Sampling information and coordinates.

Table S2 msystems.01071-26-s0003.xlsx MAG data.

Table S3 msystems.01071-26-s0004.xlsx MEBS data.

Table S4 msystems.01071-26-s0005.xlsx Dsr data.

Table S5 msystems.01071-26-s0006.xlsx Host predictions.

Table S6 msystems.01071-26-s0007.xlsx CAZyme data.

ASM does not own the copyrights to Supplemental Material that may be linked to, or accessed through, an article. The authors have granted ASM a non-exclusive, world-wide license to publish the Supplemental Material files. Please contact the corresponding author directly for reuse.
