# Supplementary material for: Shifts in vegetation impact estuary microbiomes
Source: mSystems. 2026 Sep 30;11(10):e01071-26. doi: 10.1128/msystems.01071-26 (PMC13712854; doi:10.1128/msystems.01071-26)
Supplement: Supplementary file 1 [file msystems.01071-26-s0001.pdf]

Supplementary Information

Shifts in vegetation impact estuary microbiomes

Georgina L. Aitolo[1,2*], Ian M. Rambo[3,4*], Rachel E. Weisend[5], Megan Mullis[5], Susannah Tringe[6], James Kosmopoulos[7,8], Karthik Anantharaman[8], Brandi Kiel Reese[9,10], Brett J. Baker[2,3#] and Valerie De Anda[2,3,11,#]

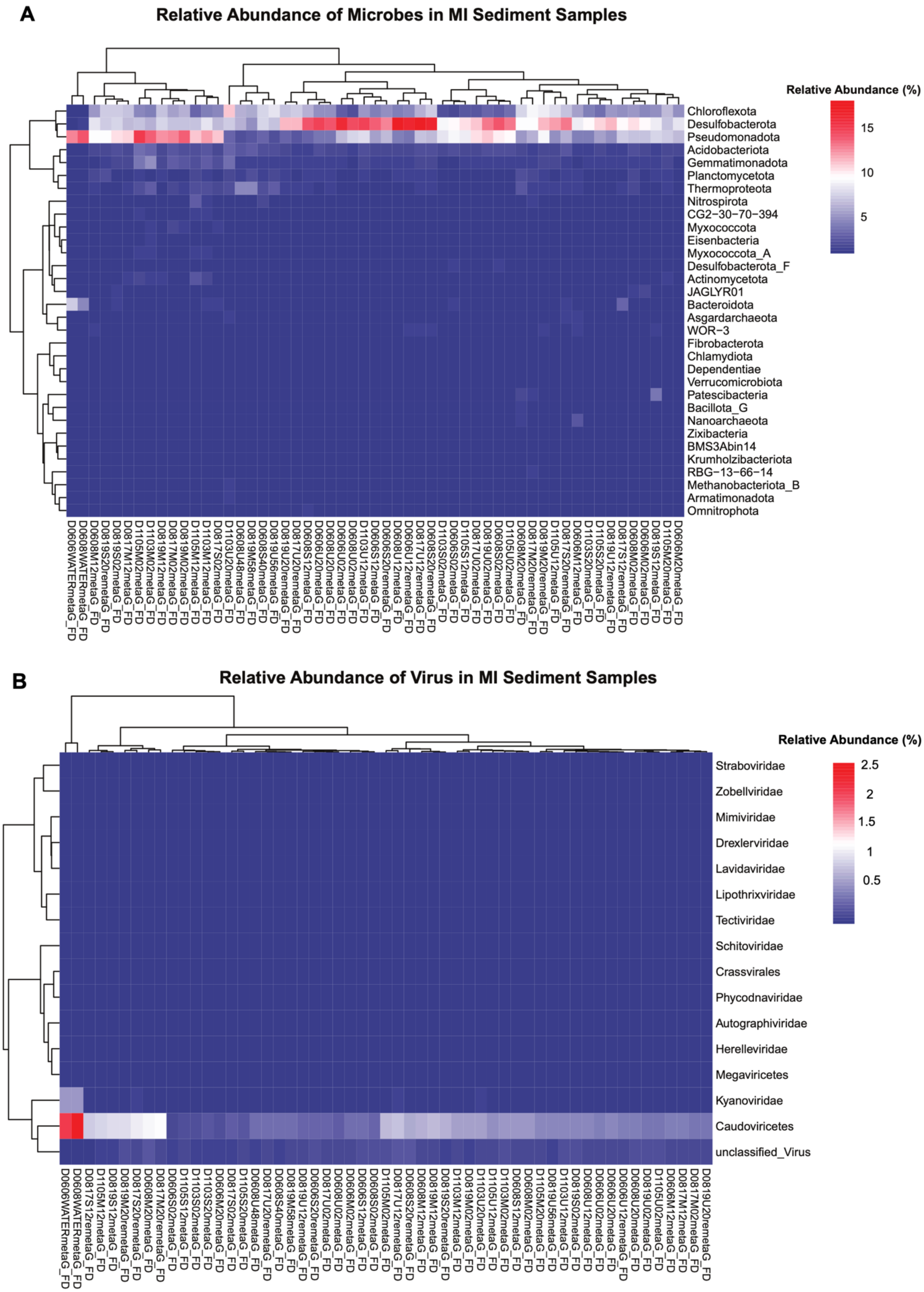
A
Relative Abundance of Microbes in MI Sediment Samples
Relative Abundance (%)
15
10
5
Chloroflexota
Desulfobacterota
Pseudomonadota
Acidobacteriota
Gemmatimonadota
Planctomycetota
Thermoproteota
Nitrospirota
CG2-30-70-394
Myxococcota
Eisenbacteria
Myxococcota_A
Desulfobacterota_F
Actinomycetota
JAGLYR01
Bacteroidota
Asgardarchaeota
WOR-3
Fibrobacterota
Chlamydiota
Dependentiae
Verrucomicrobiota
Patescibacteria
Bacillota_G
Nanoarchaeota
Zixibacteria
BMS3Abin14
Krumholzibacteriota
RBG-13-66-14
Methanobacteriota_B
Armatimonadota
Omnitrophota
B
Relative Abundance of Virus in MI Sediment Samples
Relative Abundance (%)
2.5
2
1.5
1
0.5
Straboviridae
Zobellviridae
Mimiviridae
Drexlerviridae
Lavidaviridae
Lipothrixviridae
Tectiviridae
Schitoviridae
Crassvirales
Phycodnaviridae
Autographiviridae
Herelleviridae
Megaviricetes
Kyanoviridae
Caudoviricetes
unclassified_Virus

Fig. S1. Heatmap showing the relative abundance of microbial and viral MAGs in 55 metagenomic samples across MI sediments. A. Relative abundance of 491 MAGs, summed by phylum. B. This shows the relative abundance of viruses, summed by class. The relative abundance is shown as the proportion of mapped reads to each MAGs and vMAGs, as calculated by CoverM. Unclassified viruses indicate virus sequences that reveal no taxonomic classification by genomaD. The x-axis represents the individual metagenomic samples, while the y-axis represents the taxonomic classifications of the microbial group (bacteria, archaea, and viruses).

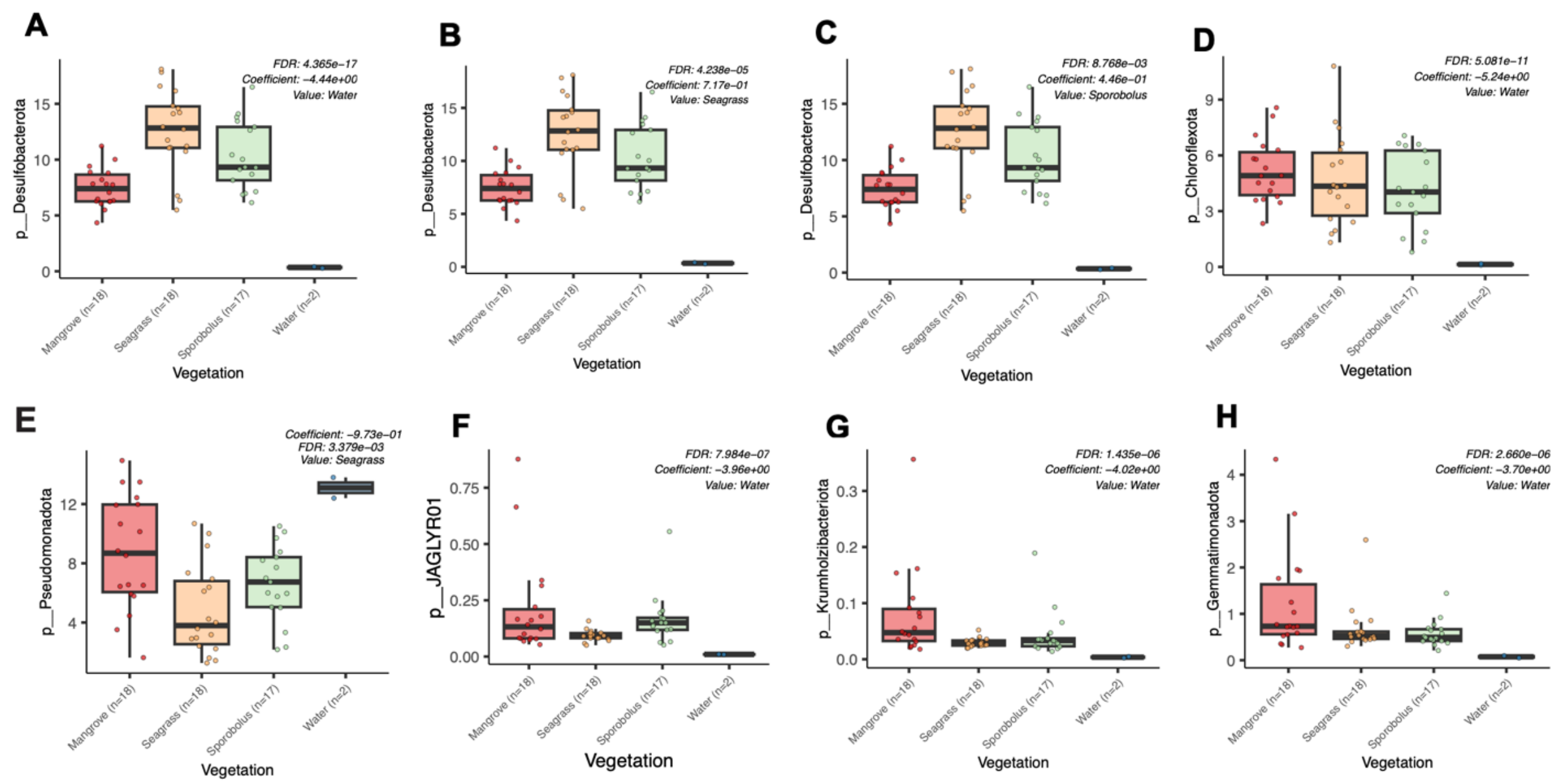

Fig. S2. Boxplot showing MaAslin2 differential abundance of microbial communities based on vegetation types. A-H The FDR (false discovery rate) values, regression coefficients and vegetation are shown on the top right corner of each plot. The statistical test was performed with the following parameters: fixed_effects = “vegetation”, normalization = “NONE”, standardize = FALSE, reference = “vegetation,mangrove”, max_significance = 0.05. The x-axis shows the individual phylum of the microbial group being tested, while the y-axis indicates relative abundance.

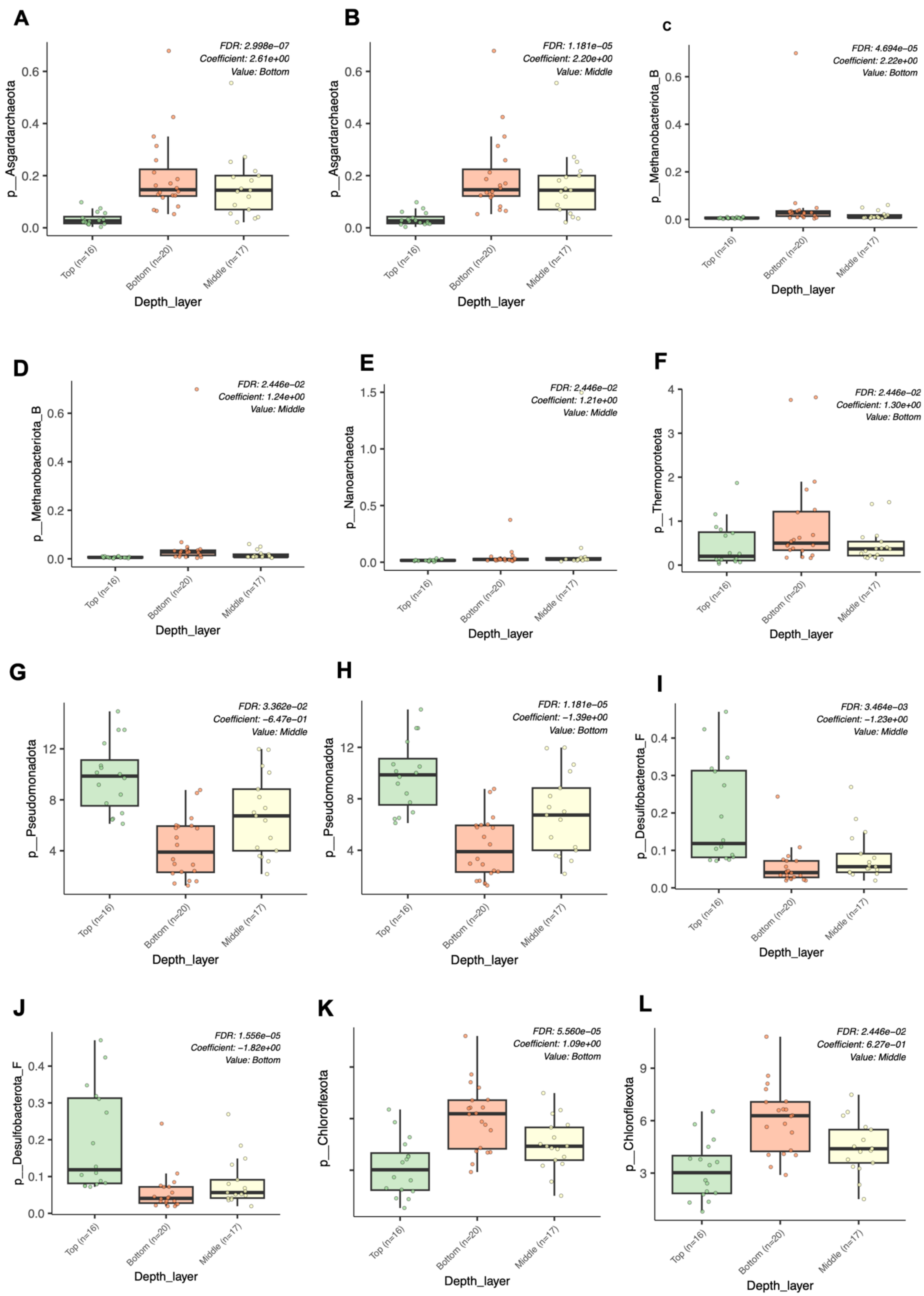
A
FDR: 2.998e−07
Coefficient: 2.61e+00
Value: Bottom
p__Asgardarchaeota
Top (n=16)
Bottom (n=20)
Middle (n=17)
Depth_layer
B
FDR: 1.181e−05
Coefficient: 2.20e+00
Value: Middle
p__Asgardarchaeota
c
FDR: 4.694e−05
Coefficient: 2.22e+00
Value: Bottom
p__Methanobacteriota_B
D
FDR: 2.446e−02
Coefficient: 1.24e+00
Value: Middle
p__Methanobacteriota_B
E
FDR: 2.446e−02
Coefficient: 1.21e+00
Value: Middle
p__Nanoarchaeota
F
FDR: 2.446e−02
Coefficient: 1.30e+00
Value: Bottom
p__Thermoproteota
G
FDR: 3.362e−02
Coefficient: −6.47e−01
Value: Middle
p__Pseudomonadota
H
FDR: 1.181e−05
Coefficient: −1.39e+00
Value: Bottom
p__Pseudomonadota
I
FDR: 3.464e−03
Coefficient: −1.23e+00
Value: Middle
p__Desulfobacterota_F
J
FDR: 1.556e−05
Coefficient: −1.82e+00
Value: Bottom
p__Desulfobacterota_F
K
FDR: 5.560e−05
Coefficient: 1.09e+00
Value: Bottom
p__Chloroflexota
L
FDR: 2.446e−02
Coefficient: 6.27e−01
Value: Middle
p__Chloroflexota

Fig. S3. Boxplot showing MaAslin2 differential abundance of microbial communities based on depth profile. A-L. The FDR (false discovery rate) values, regression coefficients and depth are shown on the top right corner of each plot. The statistical test was performed with the following parameters: fixed_effects = “depth”, normalization = “NONE”, standardize = FALSE, reference = “depth,two”, max_significance = 0.05. The x-axis indicates individual phylum of the microbial group, while the y-axis shows relative abundance at each depth.

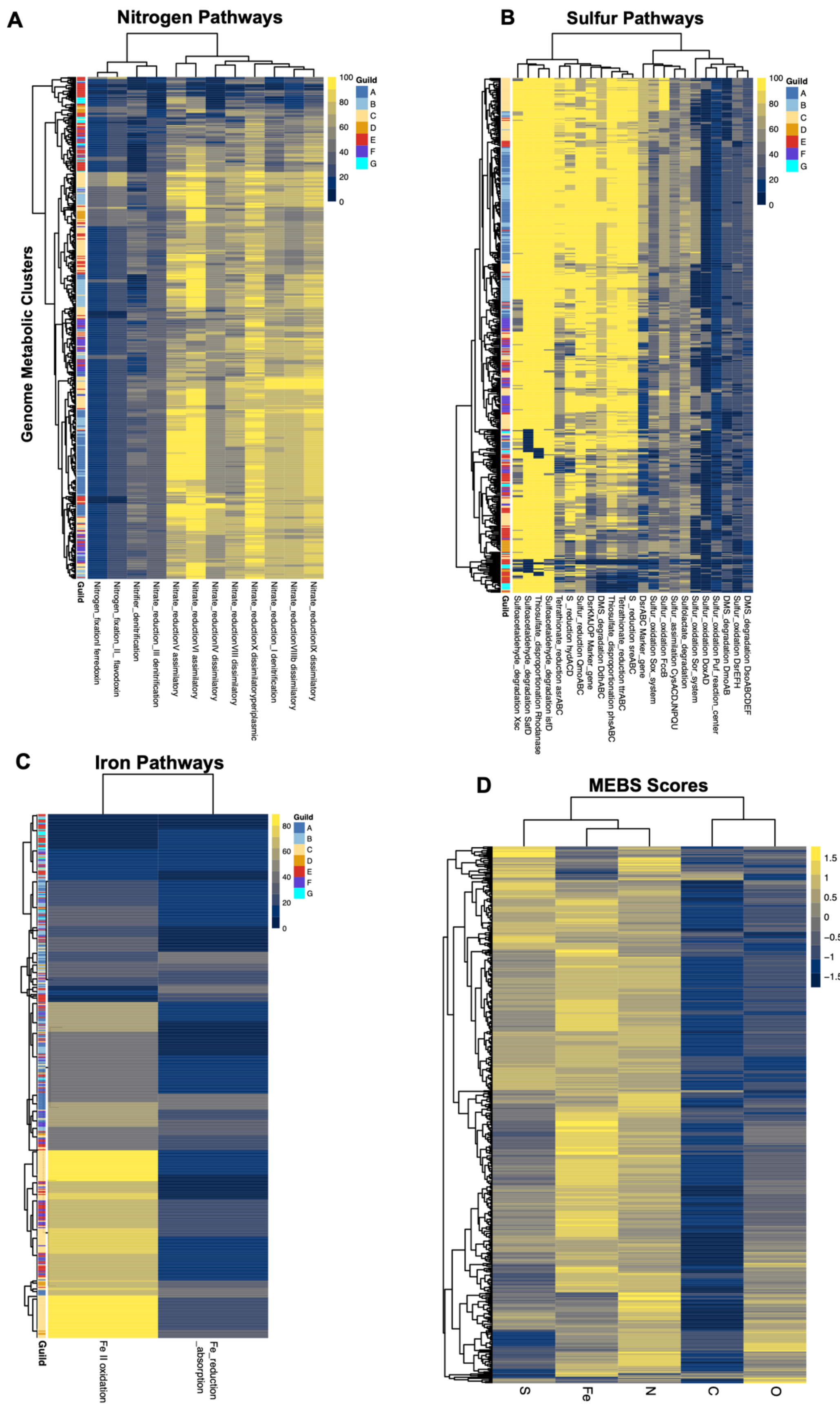
A
Nitrogen Pathways
Genome Metabolic Clusters
Guild
A
B
C
D
E
F
G
100
80
60
40
20
0
Nitrogen_fixation_ferredoxin
Nitrogen_fixation_II_flavodoxin
Nitrifier_denitrification
Nitrate_reduction_III denitrification
Nitrate_reductionV assimilatory
Nitrate_reductionVI assimilatory
Nitrate_reductionIV dissimilatory
Nitrate_reductionVIII dissimilatory
Nitrate_reductionX dissimilatoryperiplasmic
Nitrate_reduction_I denitrification
Nitrate_reductionVIIIb dissimilatory
Nitrate_reductionX dissimilatory
B
Sulfur Pathways
Sulfoacetaldehyde_degradation Xsc
Sulfoacetaldehyde_degradation SafD
Thiosulfate_disproportionation Rhodanase
Sulfoacetaldehyde_degradation isfD
Tetrathionate_reduction asrABC
S_reduction hydACD
Sulfur_reduction QmoABC
DsrKMJOP Marker_gene
DMS_degradation DdhABC
Thiosulfate_disproportionation phsABC
Tetrathionate_reduction ttrABC
S_reduction sreABC
DsrABC Marker_gene
Sulfur_oxidation Sox_system
Sulfur_oxidation FccB
Sulfur_assimilation CysACDJNPQU
Sulfolactate_degradation
Sulfur_oxidation Sor_system
Sulfur_oxidation DoxAD
Sulfur_oxidation Puf_reaction_center
DMS_degradation DmoAB
Sulfur_oxidation DsrEFH
DMS_degradation DsoABCDEF
C
Iron Pathways
80
60
40
20
0
Fe II oxidation
Fe_reduction _absorption
D
MEBS Scores
1.5
1
0.5
0
−0.5
−1
−1.5
S
Fe
N
C
O

Fig. S4. Metabolic pathway completeness and profiles of MAGs in MI sediments. A-D. The heatmap represents completeness of PFAM-based metabolic pathways (y-axis) for the set of 491 genomes. The dendrograms are representative of hierarchical clustering of genomes/guild based on pathway completeness (left), and clustering of metabolic pathways based on completeness within the genome set (bottom). Pathways on the x-axis are labeled to indicate their overall roles in nitrogen (A), sulfur (B), and iron (C) cycles. Warmer colors indicate higher percent completeness. A. Nitrogen pathway shows dissimilatory nitrate and nitrite reduction are also broadly distributed in the MI microbes. Microbial genomes in B, mainly contains sulfoacetaldehyde degradation and Rhodanase, which are enzymes involved in sulfur degradation and detoxification. C. Several MAGs in metabolic guild C encoding genes for iron (II) oxidation. D. Heatmap shows the normalized entropy scores of the MAGs based on MEBS (Multigenomic Entropy-Based Score). On the left is the hierarchical clustering of the MAGs and the top dendrogram shows the different metabolic pathways present.

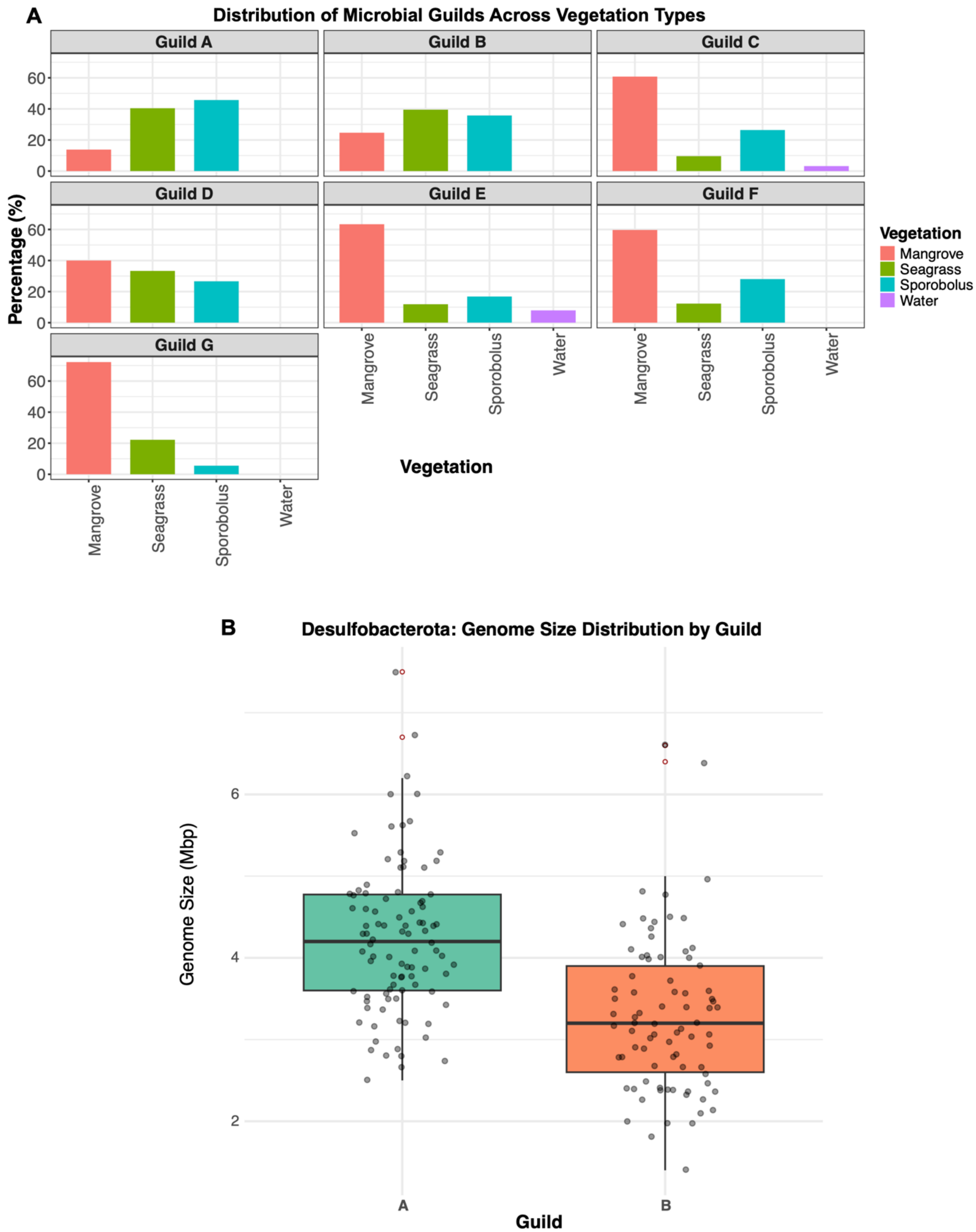
A
Distribution of Microbial Guilds Across Vegetation Types
Guild A
Guild B
Guild C
Guild D
Guild E
Guild F
Guild G
Percentage (%)
0
20
40
60
Mangrove
Seagrass
Sporobolus
Water
Vegetation
Vegetation
Mangrove
Seagrass
Sporobolus
Water
B
Desulfobacterota: Genome Size Distribution by Guild
Genome Size (Mbp)
2
4
6
A
B
Guild

Fig. S5. Metabolic guild distribution across vegetation types and genomic characteristics of Desulfobacterota. A. The bar plot represents the proportion and distribution of microbial metabolic guilds across the vegetation types. B. Boxplot shows the variation in the genome size of Desulfobacterota MAGs based on their metabolic guilds.

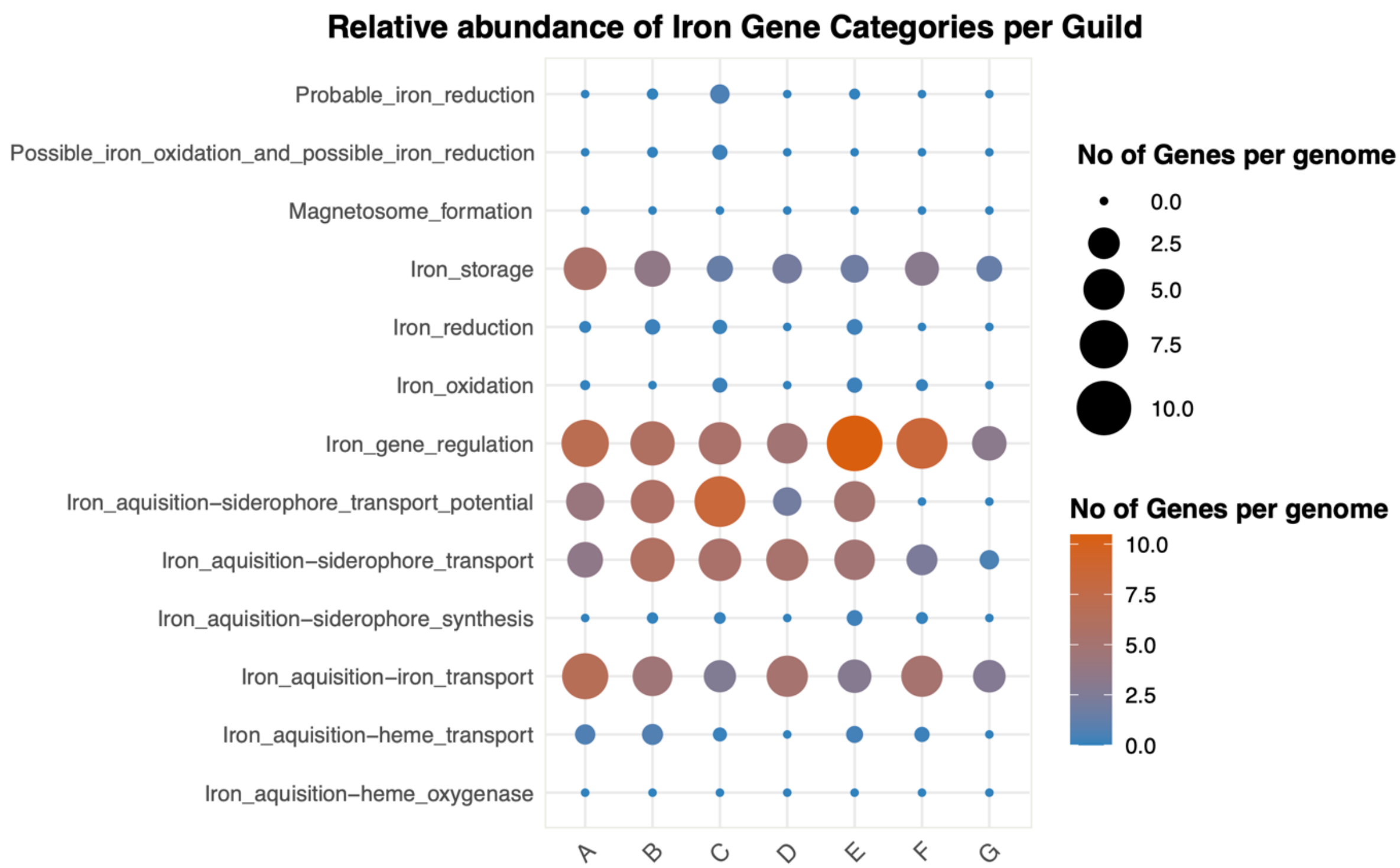

Fig. S6. Abundance of iron cycling genes across microbial metabolic guilds. Dotplot showing the relative abundance of iron gene categories grouped by the metabolic guilds according to FeGenie. The size and the color gradient of each dot reflect the normalized count of genes by the number of genomes in each phylum.

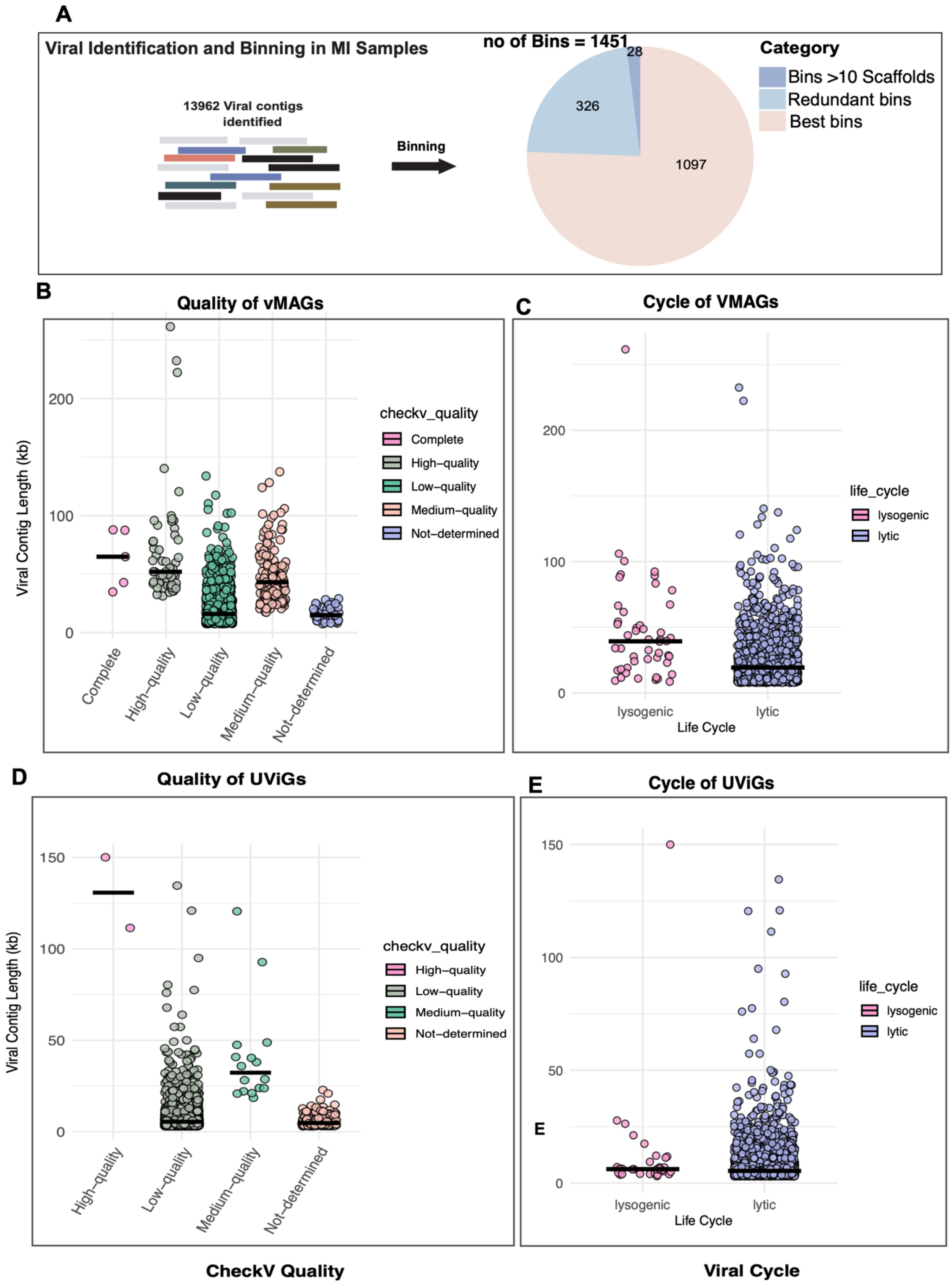
A
Viral Identification and Binning in MI Samples
13962 Viral contigs identified
Binning
no of Bins = 1451
28
326
1097
Category
Bins >10 Scaffolds
Redundant bins
Best bins
B
Quality of vMAGs
Viral Contig Length (kb)
checkv_quality
Complete
High−quality
Low−quality
Medium−quality
Not−determined
C
Cycle of VMAGs
life_cycle
lysogenic
lytic
Life Cycle
D
Quality of UViGs
Viral Contig Length (kb)
checkv_quality
High−quality
Low−quality
Medium−quality
Not−determined
E
Cycle of UViGs
life_cycle
lysogenic
lytic
Life Cycle
CheckV Quality
Viral Cycle

Fig. S7. Identification and Quality assessment of viruses in MI sediments. A. Total number of viral genomes discovered in MI sediments as determined by VIBRANT (Top-left). Pie Chart showing the number of vMAGs binned using vRhyme (Top-right). Viral (n > 10 scaffolds) and redundant bins are further broken down as scaffolds and UViGs. B. Jitter plot displays the CheckV quality of vMAGs (> 3kb length) ranging from complete viral genomes to Not-determined. C. Jitter plot showing the viral lifestyle of vMAGs categorized into lysogenic and lytic. D. UViGs quality; from high to Not-determined, E. Jitter plot displaying the viral lifestyle (lytic vs lysogenic) of UViGs.

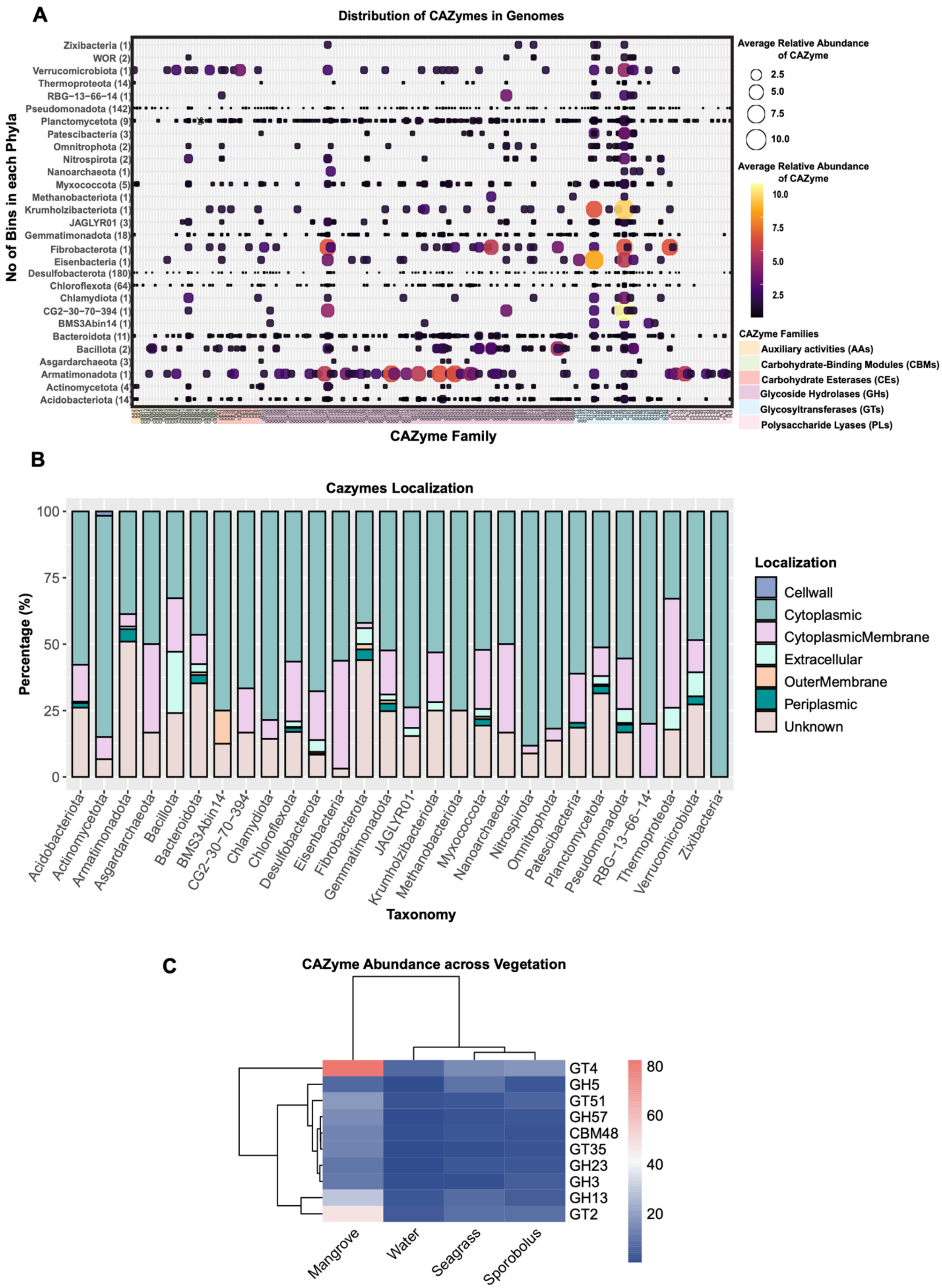
A
Distribution of CAZymes in Genomes
Zixibacteria (1)
WOR (2)
Verrucomicrobiota (1)
Thermoproteota (14)
RBG−13−66−14 (1)
Pseudomonadota (142)
Planctomycetota (9)
Patescibacteria (3)
Omnitrophota (2)
Nitrospirota (2)
Nanoarchaeota (1)
Myxococcota (5)
Methanobacteriota (1)
Krumholzibacteriota (1)
JAGLYR01 (3)
Gemmatimonadota (18)
Fibrobacterota (1)
Eisenbacteria (1)
Desulfobacterota (180)
Chloroflexota (64)
Chlamydiota (1)
CG2−30−70−394 (1)
BMS3Abin14 (1)
Bacteroidota (11)
Bacillota (2)
Asgardarchaeota (3)
Armatimonadota (1)
Actinomycetota (4)
Acidobacteriota (14)
No of Bins in each Phyla
CAZyme Family
Average Relative Abundance of CAZyme
2.5
5.0
7.5
10.0
Average Relative Abundance of CAZyme
10.0
7.5
5.0
2.5
CAZyme Families
Auxiliary activities (AAs)
Carbohydrate-Binding Modules (CBMs)
Carbohydrate Esterases (CEs)
Glycoside Hydrolases (GHs)
Glycosyltransferases (GTs)
Polysaccharide Lyases (PLs)
B
Cazymes Localization
Percentage (%)
100
75
50
25
0
Acidobacteriota
Actinomycetota
Armatimonadota
Asgardarchaeota
Bacillota
Bacteroidota
BMS3Abin14
CG2−30−70−394
Chlamydiota
Chloroflexota
Desulfobacterota
Eisenbacteria
Fibrobacterota
Gemmatimonadota
JAGLYR01
Krumholzibacteriota
Methanobacteriota
Myxococcota
Nanoarchaeota
Nitrospirota
Omnitrophota
Patescibacteria
Planctomycetota
Pseudomonadota
RBG−13−66−14
Thermoproteota
Verrucomicrobiota
Zixibacteria
Taxonomy
Localization
Cellwall
Cytoplasmic
CytoplasmicMembrane
Extracellular
OuterMembrane
Periplasmic
Unknown
C
CAZyme Abundance across Vegetation
GT4
GH5
GT51
GH57
CBM48
GT35
GH23
GH3
GH13
GT2
Mangrove
Water
Seagrass
Sporobolus
80
60
40
20

Fig. S8. Carbohydrate-active enzymes Distributions, Localization and Relative Abundance across microbial groups. A. CAZyme abundance within each microbial phylum based on dbCAN. The dot size and color intensity represent the average abundance of CAZymes per phylum, calculated as the total CAZyme count divided by the number of MAGs within that phylum**.** B. CAZymes subcellular localization and percentage distribution within each microbial phylum. C. Heatmap showing the abundance of the top 10 CAZyme families associated with different vegetation types.

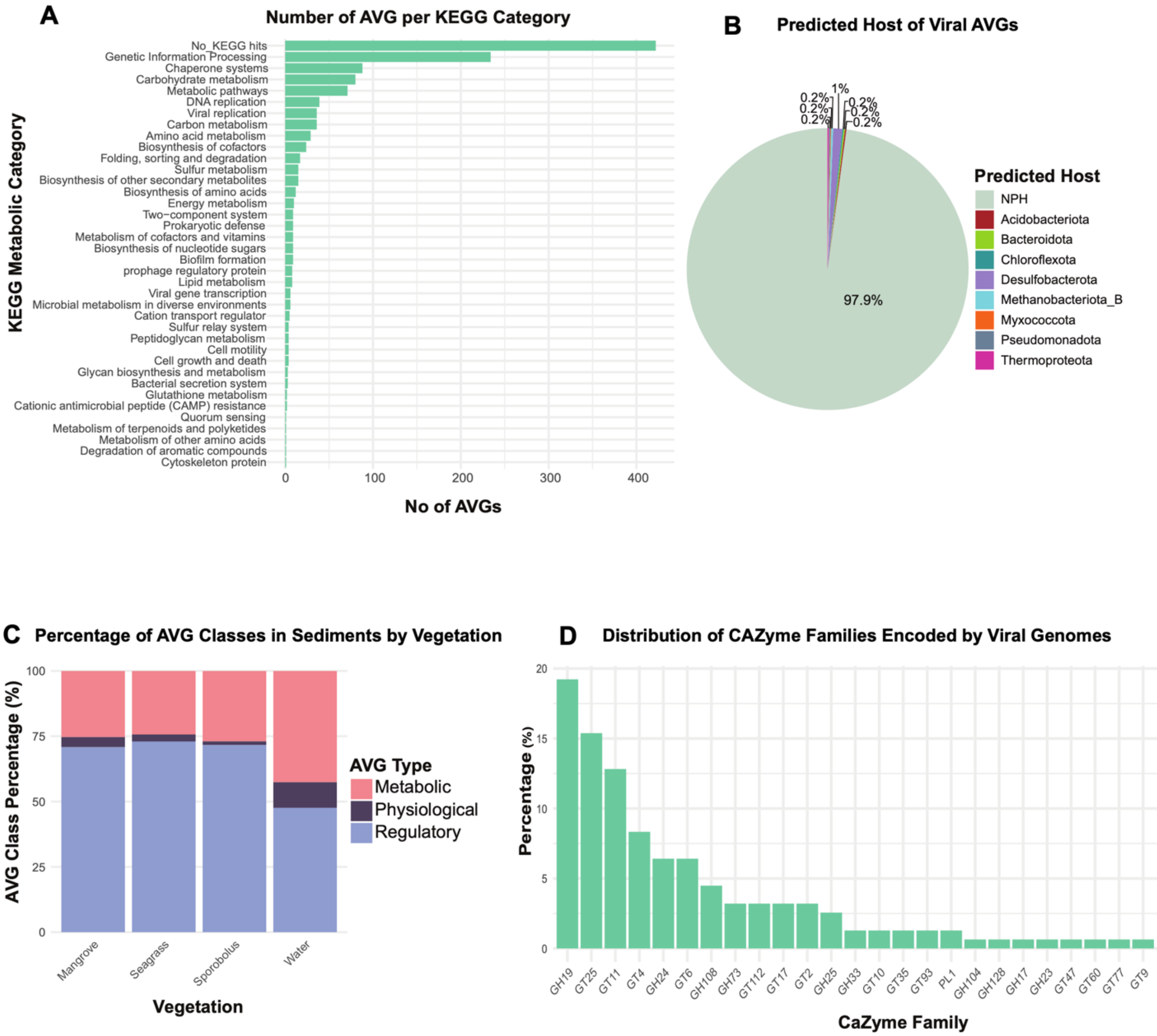

Fig. S9. Identification of AVGs and Distribution of Viral CAZymes in MI sediments. A. Classification of viral-encoded AVGs in viral genomes based on KEGG metabolic categories. B. Percentage composition of predicted host taxa for AVGs. NPH refers to No-predicted-Host. C. Stacked bar plot represents the percentage of AVG types across different vegetation types. D. Bar plot shows the relative abundance and distribution of CAZyme families in viruses. The CAZyme families are grouped into six categories: AA (auxiliary activities), CBM (carbohydrate-binding module), CE (carbohydrate esterases), GH (glycoside-hydrolases), GT (glycoside transferases), PL (polysaccharide lyases).
